# Hypomorphic mutations of *TRIP11* cause odontochondrodysplasia

**DOI:** 10.1172/jci.insight.124701

**Published:** 2019-02-07

**Authors:** Anika Wehrle, Tomasz M. Witkos, Sheila Unger, Judith Schneider, John A. Follit, Johannes Hermann, Tim Welting, Virginia Fano, Marja Hietala, Nithiwat Vatanavicharn, Katharina Schoner, Jürgen Spranger, Miriam Schmidts, Bernhard Zabel, Gregory J. Pazour, Agnes Bloch-Zupan, Gen Nishimura, Andrea Superti-Furga, Martin Lowe, Ekkehart Lausch

**Affiliations:** 1 Department of Pediatrics, Medical Center-University of Freiburg, Faculty of Medicine, University of Freiburg, Freiburg, Germany.; 2 Faculty of Biology, Medicine and Health, University of Manchester, Manchester, United Kingdom.; 3 Division of Genetic Medicine, University of Lausanne, Centre Hospitalier Universitaire Vaudois, Lausanne, Switzerland.; 4 Program in Molecular Medicine, University of Massachusetts Medical School, Worcester, Massachusetts, USA.; 5 Laboratory for Experimental Orthopedics, Department of Orthopaedic Surgery, Maastricht University Medical Centre, Maastricht, the Netherlands.; 6 Hospital de Pediatria JP Garrahan, Buenos Aires, Argentina.; 7 Medical Biochemistry and Genetics, University of Turku, Turku, Finland.; 8 Department of Paediatrics, Mahidol University, Bangkok, Thailand.; 9 Institute of Pathology, Philipps-University Marburg, Marburg, Germany.; 10 Centre de Référence des Manifestations Odontologiques des Maladies Rares, Pôle de Médecine et Chirurgie Bucco-dentaires, Hôpitaux Universitaires de Strasbourg (HUS), Faculté de Chirurgie Dentaire, Université de Strasbourg, Strasbourg, France.; 11 Université de Strasbourg, Faculté de Chirurgie Dentaire, Institute of Advanced Studies, USIAS, Strasbourg, France.; 12 HUS, Pôle de Médecine et Chirurgie Bucco-dentaires Hôpital Civil, Centre de référence des maladies rares orales et dentaires, O-Rares, Filière Santé Maladies rares TETE COU, European Reference Network ERN CRANIO, Strasbourg, France.; 13 Université de Strasbourg, Institut de Génétique et de Biologie Moléculaire et Cellulaire (IGBMC), CERBM, INSERM U1258, CNRS- UMR7104, Illkirch, France.; 14 Department of Radiology and Medical Imaging, Tokyo Metropolitan Kiyose Children’s Hospital, Kiyose, Japan.

**Keywords:** Bone Biology, Genetics, Bone development, Molecular pathology, Protein traffic

## Abstract

Odontochondrodysplasia (ODCD) is an unresolved genetic disorder of skeletal and dental development. Here, we show that ODCD is caused by hypomorphic *TRIP11* mutations, and we identify ODCD as the nonlethal counterpart to achondrogenesis 1A (ACG1A), the known null phenotype in humans. *TRIP11* encodes Golgi-associated microtubule-binding protein 210 (GMAP-210), an essential tether protein of the Golgi apparatus that physically interacts with intraflagellar transport 20 (IFT20), a component of the ciliary intraflagellar transport complex B. This association and extraskeletal disease manifestations in ODCD point to a cilium-dependent pathogenesis. However, our functional studies in patient-derived primary cells clearly support a Golgi-based disease mechanism. In spite of reduced abundance, residual GMAP variants maintain partial Golgi integrity, normal global protein secretion, and subcellular distribution of IFT20 in ODCD. These functions are lost when GMAP-210 is completely abrogated in ACG1A. However, a similar defect in chondrocyte maturation is observed in both disorders, which produces a cellular achondrogenesis phenotype of different severity, ensuing from aberrant glycan processing and impaired extracellular matrix proteoglycan secretion by the Golgi apparatus.

## Abstract 2

Bi-allelic mutations of TRIP11 cause a spectrum of skeletal phenotypes whose severity is primarily based on impaired secretory trafficking and aberrant glycan processing.

## Graphical abstract

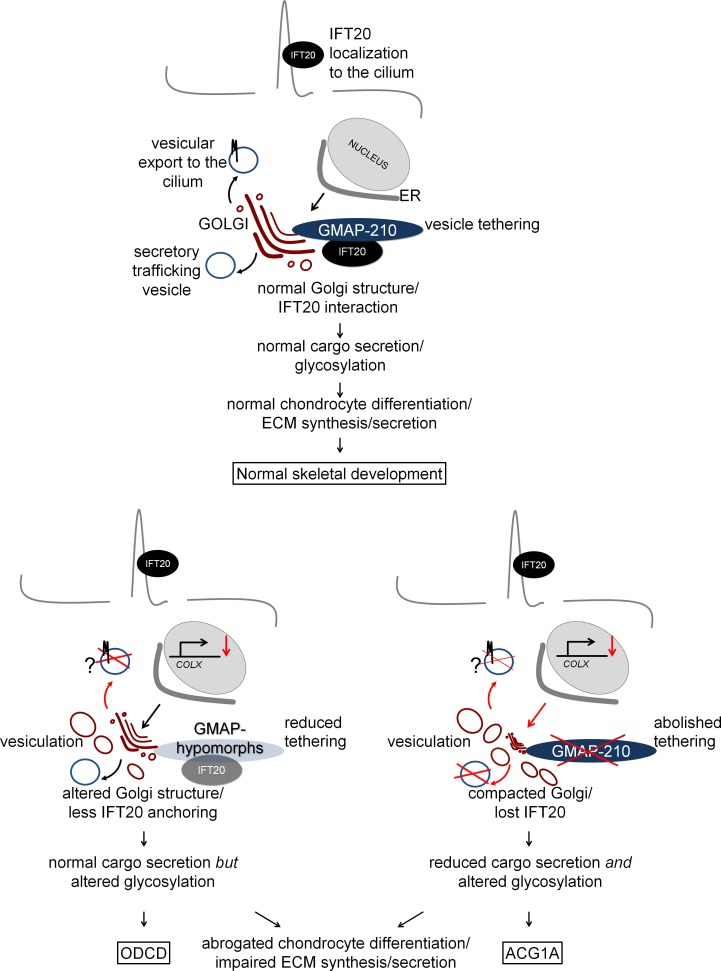

## Introduction

Thyroid hormone receptor interactor 11 (TRIP11, also known as Golgi-associated microtubule-binding protein 210 [GMAP-210], MIM 604505) is essential for normal skeletal development and endochondral ossification. Previously, recessive loss-of-function mutations of the *TRIP11* gene were found to cause achondrogenesis type 1A (ACG1A, MIM 200600) (1), a severe chondrodysplasia characterized by short trunk, narrow chest, short extremities, and craniofacial malformations (2, 3). In the majority of cases, antenatal suspicion of a lethal chondrodysplasia based on sonographic findings leads to an early termination of pregnancy (4). In ACG1A fetuses who are carried to full term, thoracic hypoplasia and rib fractures lead to respiratory insufficiency and perinatal death (3). Respiratory failure and perinatal death were also observed in mice with a homozygous-targeted deletion of *Trip11* (1, 5, 6). Lethality in mice may, however, may also be due to primary pulmonary pathology rather than a small rib cage, which is considered the critical problem in humans (5). Characterization of the skeletal phenotype and functional studies in mice suggested that the pathogenesis of ACG1A may be explained by the known intracellular function of GMAP-210 as a Golgi-associated vesicle tethering protein (1, 6–8). Conversely, GMAP-210 interacts with intraflagellar transport 20 (IFT20) that is involved in ciliary trafficking processes and phenotypic features in both mice and humans, such as thoracic dystrophy, pulmonary dysplasia, and hydrocephaly, suggesting that developmental defects in ACG1A may also be due to impaired ciliary functions (5, 9).

Odontochondrodysplasia (ODCD, MIM 184260) is an unresolved skeletal dysplasia recognized as a distinct entity by Goldblatt et al. in 1991 (10). Key clinical findings are short stature, narrow chest, mesomelic limb shortening, brachydactyly, joint laxity, and dental anomalies (11). Radiographic features include platyspondyly with coronal clefts and metaphyseal irregularities of the tubular bones (12).

We here unravel the molecular basis of ODCD and describe a genotype-phenotype correlation, ranging from ACG1A as the null phenotype to ODCD caused by recurrent hypomorphic *TRIP11* mutations. Detailed analyses of *TRIP11*-mutant primary cells revealed that Golgi organization, global secretory capacity, and IFT20 Golgi targeting are preserved in ODCD but are lost in ACG1A. However, in both mild and severe disease, mutations of *TRIP11* impair Golgi glycan processing and synthesis of glycosylated cartilage matrix proteins, specifically disrupting hypertrophic chondrocyte differentiation in skeletal development.

## Results

### The clinical presentation of ODCD is variable and includes renal and cerebral anomalies.

To unravel the genetic basis of ODCD, we ascertained a series of 10 patients from 7 unrelated families; cases 1–6 were published previously (Table 1, patient and family numbering corresponds to the report of Unger et al., 2008; ref. 12). All additional index cases met the clinical and radiographic criteria defined earlier (11, 12). Clinical follow-up of the published and new families further contributed important information (Table 1). First, pedigrees supported recessive inheritance (Supplemental Figure 1; supplemental material available online with this article; https://doi.org/10.1172/jci.insight.124701DS1). Second, additional extraskeletal disease manifestations of ODCD included pulmonary dysplasia, cystic renal disease, and nonobstructive hydrocephaly. Third, it became evident that disease manifestations may range from early lethal to long-term survival with short stature (Table 1).

**Figure 1:**
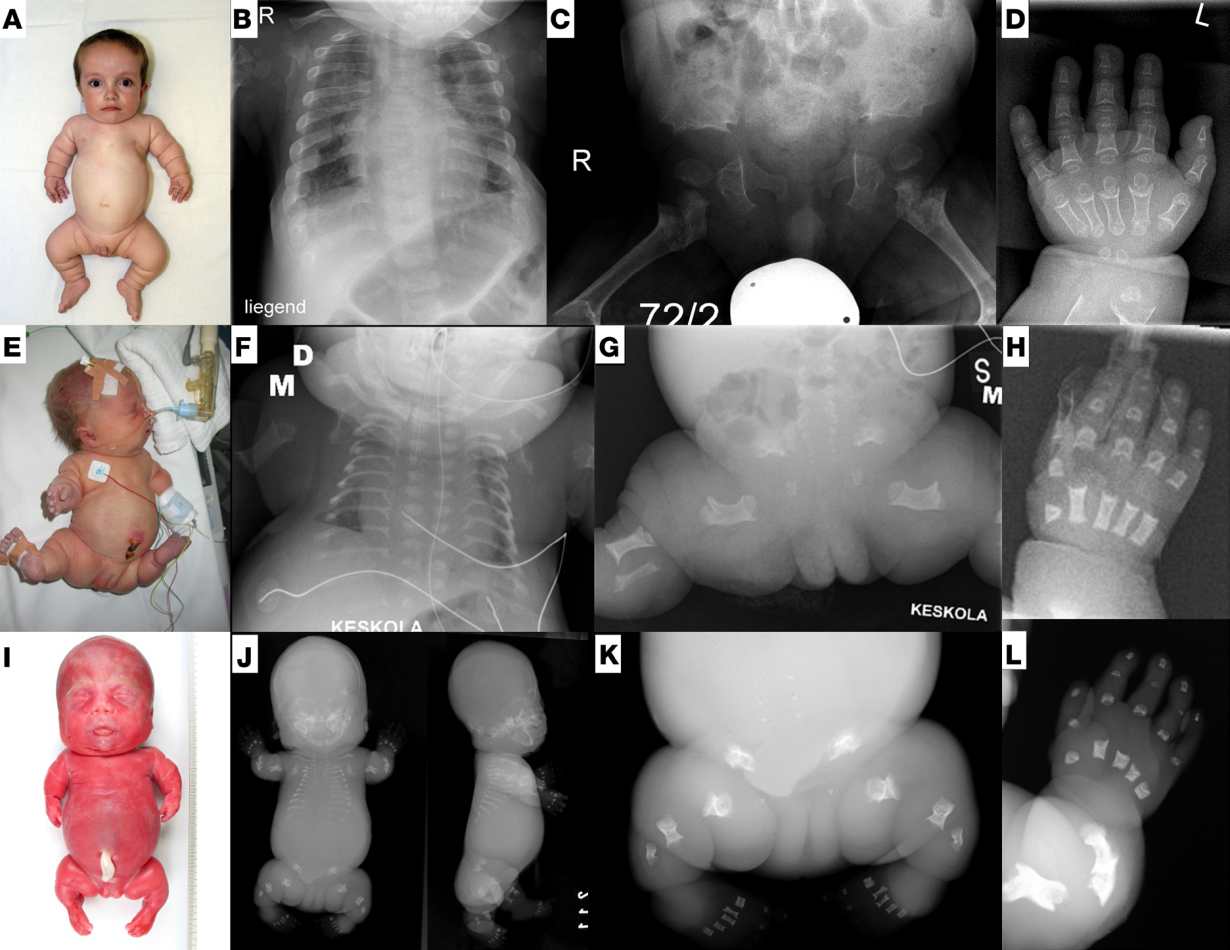
Clinical and radiographic spectrum of *TRIP11*-related disorders. (**A–D**) Odontochondrodysplasia, case 6 at age 2 years; radiographs were obtained at the age of 3 years. (**E–H**) Mild achondrogenesis 1A (ACG1A); case A3 at day 3 of life. (**I–L**) Severe ACG1A; case A5 at 22 weeks of gestation. (**B**, **F**, and **J**) Radiographs demonstrate thoracic dystrophy of increasing severity; horizontally oriented short ribs have splayed ends. Vertebral bodies are flat, clefted, and under-ossified. (**C**, **G**, and **K**) Iliac bones are hypoplastic; ossification of ischial and pubic bones is strongly retarded or absent. Tubular bones are extremely short and bowed with metaphyseal cupping. (**D**, **H**, and **L**) Severe brachydactyly of metacarpals and phalanges, which show metaphyseal cupping and bilateral spurs of cortical bone extending longitudinally.

On review of the early radiographic presentation of ODCD, we realized close similarities between ACG1A and severe ODCD (Figure 1). In both disorders, there was intra- and interfamilial clinical and radiographic variability (13). Furthermore, some alterations such as short, plump tubular bones with cupped metaphyses flanked by longitudinal spurs, short ribs, and a trident pelvis supported the assumption of a skeletal ciliopathy (14). We therefore screened for ciliopathic disease manifestations in ODCD. Clinical characteristics of ciliopathies, such as renal congenital hypodysplasia, childhood-onset cystic kidney degeneration and relative macrocephaly, were found in a few patients (Table 1) (14), in support of a possibly cilium-based pathophysiology (15). An important consequence of our observations for the clinical management of ODCD is the need for regular screening, including neuroimaging and ophthalmoscopy, to prevent secondary complications from hydrocephaly and unrecognized renal failure.

### Splice mutations of TRIP11 cause ODCD.

**Figure 2:**
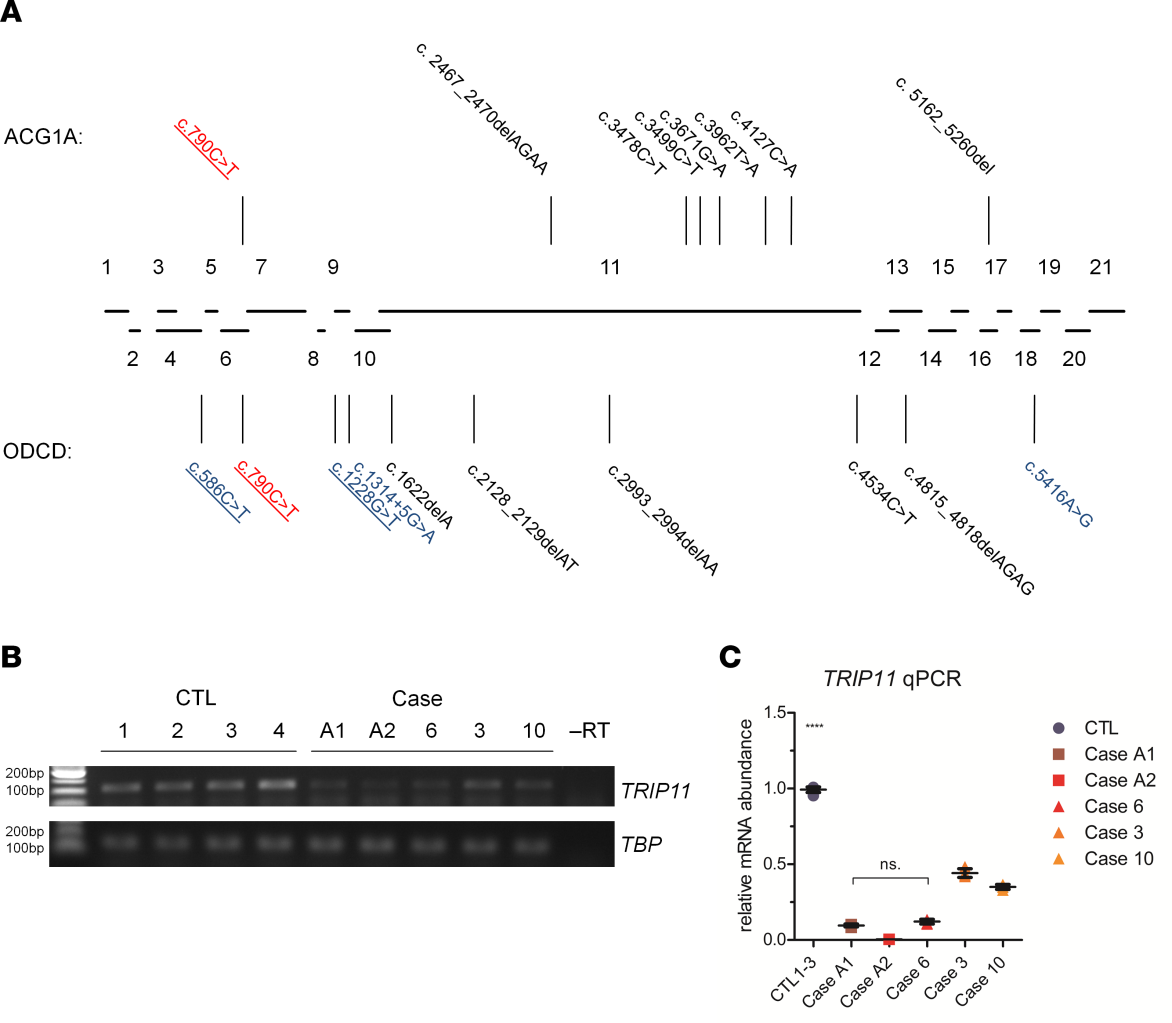
Recessive mutations in *TRIP11* cause achondrogenesis type 1A (ACG1A) and odontochondrodysplasia (ODCD). (**A**) Diagram of the human *TRIP11* locus and relative size of its 21 coding exons. Lines indicate mutations identified in ACG1A (top) and ODCD (bottom) pointing to their locations within the exons. Nonsense and frameshift mutations are depicted in black, splice mutations in blue, and mutations shared by ODCD and ACG1A in red; recurrent mutations are underlined. (**B**) Semiquantitative reverse transcription PCR of *TRIP11* using cDNA of control fibroblasts (CTL1 to -4), fibroblasts of ACG1A cases A1 and A2, and ODCD cases 6, 3, and 10. *TBP* was used for normalization. (**C**) Quantitative PCR (qPCR) analysis of *TRIP11* using cDNA derived from control and patients’ fibroblasts. *TBP* and *HPRT* were used for normalization. For qPCR results, the average value of the controls (*n* = 12) was set to 1. For *TRIP11*-mutant cells, horizontal lines represent the mean of quadruplicates (*n* = 4); error bars indicate SD. Statistical differences were assessed by 2-way ANOVA with Bonferroni’s post hoc test; ****P < 0.0001. –RT, without reverse transcriptase.

The underlying genetic defect of ACG1A has been identified as recessive loss-of-function of the *TRIP11* gene (1), which encodes GMAP-210 (16, 17). The phenotypic similarities prompted us to perform direct mutation analysis of *TRIP11* in ODCD. In all affected individuals, Sanger sequencing revealed biallelic changes that cosegregated with the disease phenotype (Figure 2A, Supplemental Figure 1, and Table 2). Parents were heterozygous; unaffected siblings were heterozygous or WT. None of the changes were known polymorphisms or were listed in public exome databases. As in ACG1A, ODCD-associated *TRIP11* mutations were scattered over the whole gene (Figure 2A). At first glance, the mutational spectrum of *TRIP11* in ODCD was similar to ACG1A, comprising predominantly small deletions and point mutations (Figure 2A, Table 2, and Supplemental Table 1). These cause a frameshift and/or a premature stop codon in the *TRIP11* open reading frame (ORF) and, thus, predict a complete loss of protein function. One of the stop mutations in ODCD was recurrent and previously reported in ACG1A (c.790C>T) (1, 13). However, only in ODCD patients, compound heterozygous mutations were detected, which predict amino acid substitutions of the GMAP-210 protein (p.Asp410Tyr, p.Met1806Val). Furthermore, a splice mutation was found in family 1 (c.1314+5G>A, Table 2). As altered splicing may vary in different cell types and may not be fully penetrant, we hypothesized that ODCD-associated *TRIP11* mutations were less damaging at the molecular level.

For 4 ODCD patients and 2 ACG1A fetuses, about whom findings have been previously published (1), materials were available to investigate the consequences of individual mutations in detail (Table 3). As expected, mutations that introduce translational termination codons had the same effect on *TRIP11* mRNA, which was reduced to very low levels in both ACG1A and ODCD patients, most likely due to nonsense-mediated mRNA decay (NMD) (Figure 2, B and C, and Table 3) (18).

**Figure 3:**
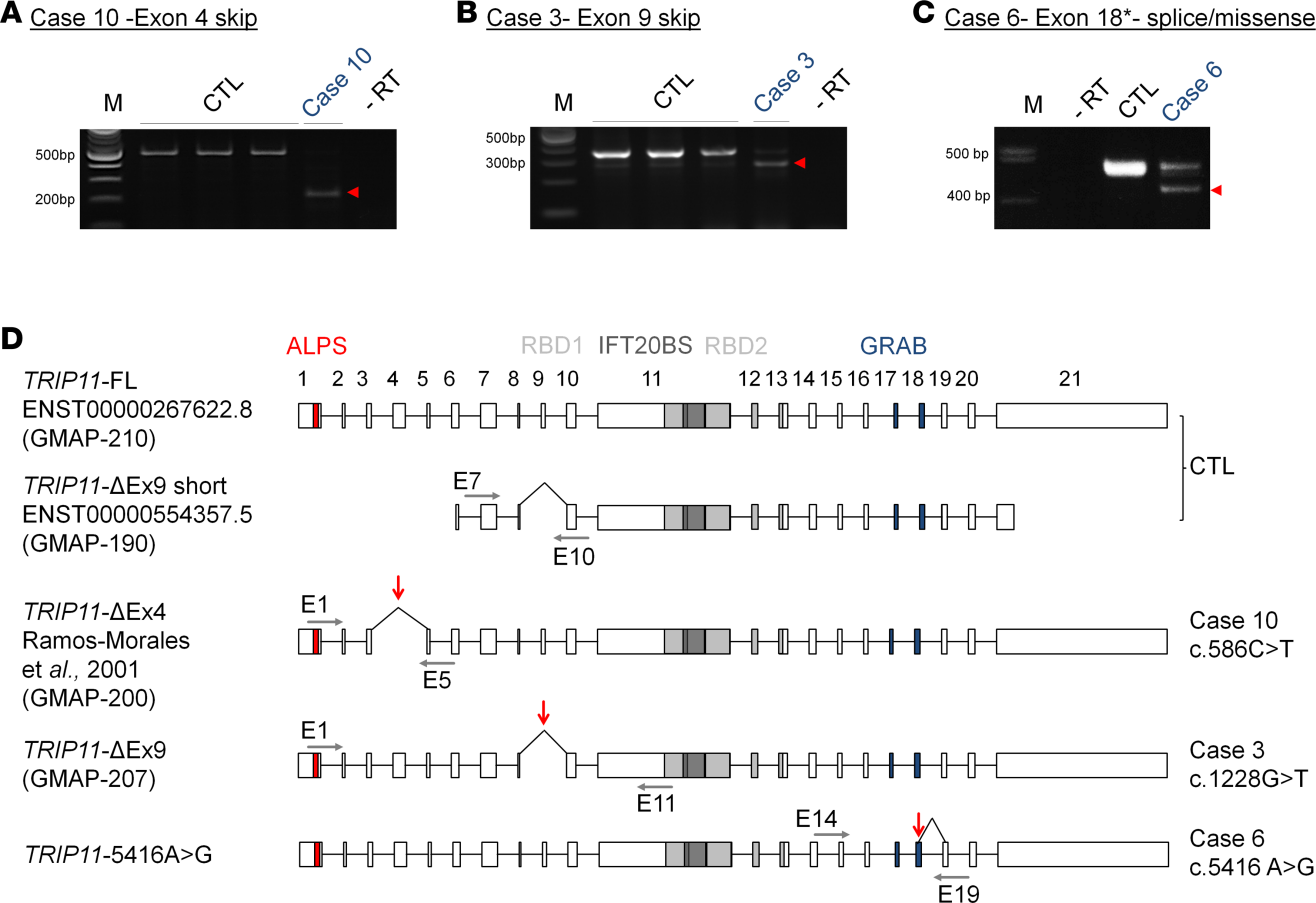
Aberrant splicing of *TRIP11* in odontochondrodysplasia. (**A–C**) Exon-spanning reverse transcription PCR (RT-PCR) analysis of *TRIP11* using cDNA derived from patient and control (CTL) fibroblasts; amplicons resulting from aberrantly spliced mRNA are marked by red arrowheads. (**A**) The c.586C>T *TRIP11* mutation causes in-frame skipping of exon 4, as indicated by the reduced PCR product size of 229 bp in case 10. (**B**) The recurrent c.1228G>T mutation causes in-frame deletion of exon 9, as indicated by the 87-bp shorter PCR product in case 3. (**C**) The c.5416A>G mutation generates an ectopic splice donor site within exon 18, as indicated by a 46-bp shorter PCR product in case 6. In addition, a PCR product of similar intensity indicating regular splicing is visible at 475 bp. (**D**) Schematic representation of *TRIP11* transcript variants including their 5′ and 3′ untranslated region detected in control (CTL) and patient fibroblasts (cases 10, 3, and 6). Exons 1–21 are shown as boxes, the size of the box correlates to the size of each exon; introns represented as lines are not to scale. Triangular-shaped lines above indicate splicing. Gray horizontal arrows indicate the relative position of primers (E1, E5, E7, E10, E11, E14, and E19) used in this study. Red vertical arrows indicate mutations. FL, full-length transcript. Amphipathic lipid sensor (ALPS) in red, which participates in vesicle tethering with an overlapping second motif (not shown); Rab-binding domains 1 and 2 (RBD1 and RBD2) in light gray, which mediate RAB2 binding; and the GRIP-related Arf-binding (GRAB) domain in blue, which mediates membrane anchoring of GMAP. The IFT20 binding site (IFT20 BS) in black is necessary for Golgi targeting of IFT20.

The recurrent c.586C>T transition, detected in 3 ODCD families, was also expected to cause NMD due to an early GMAP-210 truncation (p.Gln196*). However, we were surprised to find that the mutation, instead, abrogates a splice donor site, causing complete in-frame skipping of exon 4 (*TRIP11*-ΔEx4) in fibroblasts (case 10; Figure 3, A and D, and Supplemental Figure 2A). We therefore systematically analyzed all ODCD-associated mutations for a potential effect on splicing. Both predicted missense variants caused aberrant splicing, as well (Figure 3, B–D, and Supplemental Figure 2, B and C). They led either to in-frame skipping of exon 9 (case 3, *TRIP11*-ΔEx9) or to an exonic missplice of exon 18 (case 6). However, quantitative sequence analysis of exon-spanning cDNA amplicons in case 6 also revealed the c.5416A>G transition encoding the conservative p.Met1806Val substitution in 56% of transcripts (Supplemental Figure 3, A and B). Furthermore, while *TRIP11*-ΔEx9 including the complete 5′-end was the predominant transcript in case 3 (Supplemental Figure 3C), exon 9–containing mRNA was also found, which indicates that aberrant splicing was not fully penetrant for all mutations (Supplemental Figure 3D). The effect of splice mutations was cell type dependent, as demonstrated by a different splicing pattern in leukocytes (Supplemental Figure 4).

Taken together, the experimental evidence shows aberrant splicing of *TRIP11* in all ODCD cases investigated (Table 3). Due to recurrence of splice mutations, potentially functional GMAP proteins were predicted in all patients that are identical to, or overlapping with, natural isoforms (Figure 3D). Since splicing of *TRIP11* was cell type dependent and not fully penetrant, we assumed that transcripts in some tissues are occasionally spliced correctly and that residual missense or WT GMAP-210 protein may influence the overall phenotypic outcome.

### TRIP11 is alternatively spliced in chondrogenic differentiation.

The occurrence of in-frame *TRIP11* variants in ODCD is intriguing, since — similar to *TRIP11*-ΔEx4 (19) — transcripts lacking exon 9 and carrying a shorter 5′-end also exist in healthy individuals (*TRIP11*-ΔEx9short; Figure 3D). The cDNA of this natural isoform predicts a 190 kDa GMAP protein lacking the amino-terminus, which encompasses the amphipathic lipid-packing sensor (ALPS) motif essential for vesicle tethering (7, 20) and a recently described second vesicle tethering motif (21). Public transcriptome data suggest that *TRIP11*-ΔEx9short is transcribed in chondrocytes and osteoblasts (22, 23). We therefore performed exon-spanning reverse transcription PCR (RT-PCR) analysis of human chondrogenic cells derived from primary fibroblasts by reprogramming (24, 25). While the *TRIP11*-ΔEx4 transcript was not detectable, *TRIP11*-ΔEx9short and full-length mRNA were highly expressed (Supplemental Figure 5A). The abundance of both isoforms increased continuously during chondrogenic differentiation and was highest when cells reached the stage of hypertrophy (Supplemental Figure 5A). The same distribution of *TRIP11* mRNA isoforms was found in primary human articular chondrocytes from healthy donors (Supplemental Figure 5B).

In addition to an intense signal at 230 kDa representing the full-length GMAP-210 protein, a number of smaller GMAP proteins were detected in chondrogenic cells by using a carboxy-terminal antibody, including approximately 30 kDa lower bands present in WT fibroblast-derived chondrocytes (FDCs) (Supplemental Figure 5C). This GMAP variant, with a calculated molecular weight of 190 kDa, runs at approximately 210 kDa and may represent the translation product of *TRIP11*-ΔEx9short. In accordance with RNA analysis, both GMAP-190 and GMAP-210 proteins were induced upon chondrogenic differentiation (Supplemental Figure 5C). Thus, specific mRNA and corresponding protein isoforms are potentially relevant for endochondral ossification and skeletal development. Assuming that disease-associated and/or natural GMAP proteins may rescue embryonic lethality in ODCD, we investigated the functioning of these GMAP variants in detail.

### Shorter GMAP proteins are produced at low levels in ODCD.

**Figure 4:**
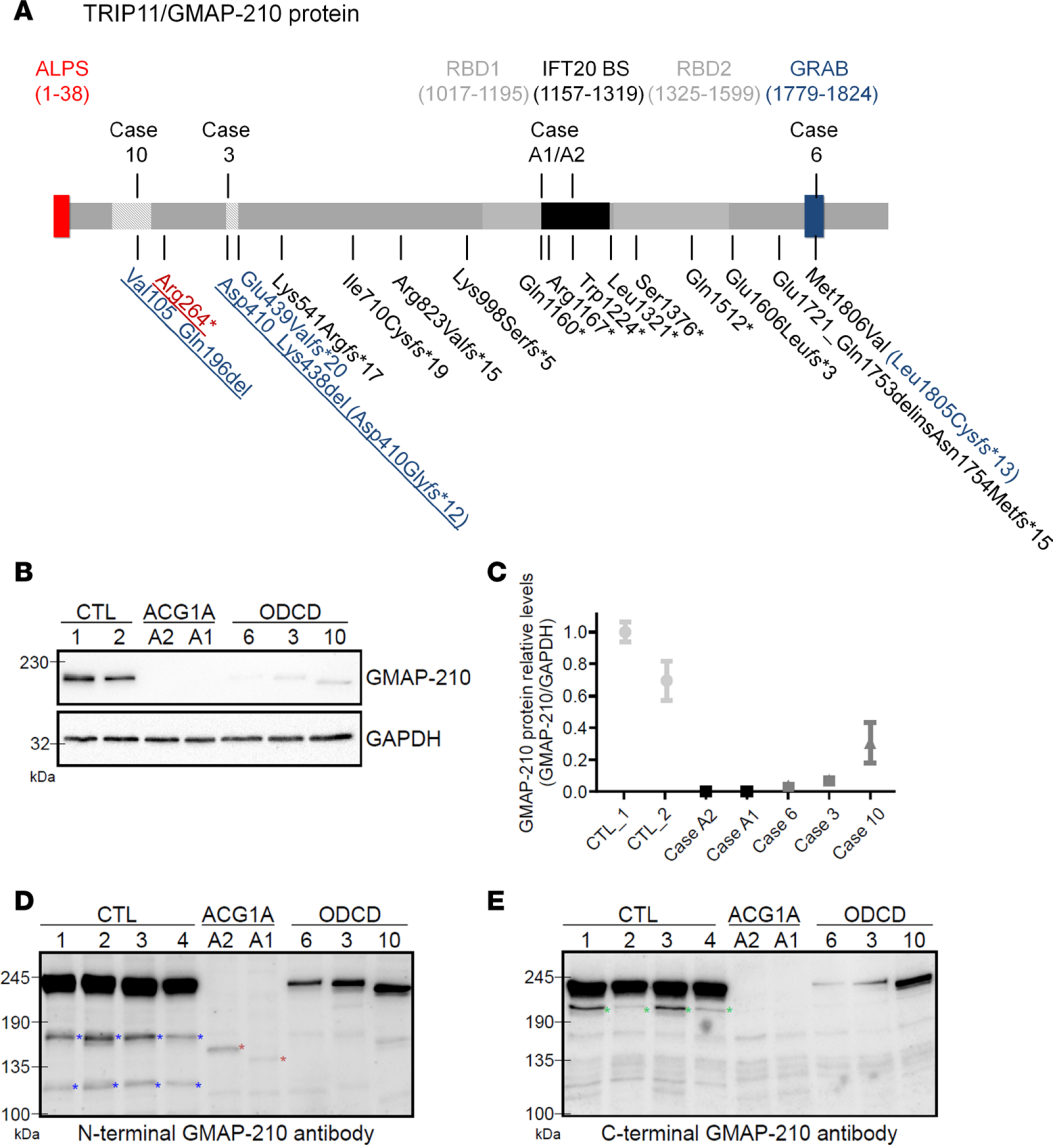
GMAP-210 protein analysis in achondrogenesis 1A (ACG1A) and odontochondrodysplasia (ODCD) patient–derived primary cells. (**A**) Schematic of the 1,979 amino acid GMAP-210 protein. The backbone of coiled-coil domains is shown in gray; the amphipathic lipid sensor (ALPS) in red, which participates in vesicle tethering with an overlapping second motif (not shown); Rab-binding domains 1 and 2 (RBD1 and RBD2) in light gray, which mediate RAB2 binding; and the GRIP-related Arf-binding (GRAB) domain in blue, which mediates membrane anchoring of GMAP. The IFT20 binding site (IFT20 BS, in black) is necessary for Golgi targeting of IFT20. Mutations identified in 5 ACG1A and 10 ODCD cases are indicated below; those amenable to functional analysis are indicated above. Amino acids deleted by in-frame exon skipping are hatched; nonsense and frameshift mutations are depicted in black, splice mutations in blue, and mutations shared by ACG1A and ODCD in red; recurrent mutations are underlined. (**B**) GMAP proteins in whole-cell protein lysates of patients and controls; GAPDH staining demonstrates total protein loading. The mean of triplicate quantitative blot signal analyses is represented in **C**; error bars indicate ± SEM. (**D–E**) GMAP-210 in whole-cell protein lysates of patients and controls using more sensitive enhanced chemiluminescence (ECL) substrate to analyze low-abundant GMAP protein variants with polyclonal antibodies directed against the N-terminus and the C-terminus. (**D**) Note the ~3 kDa lower protein variant in case 3 (consistent with the loss of exon 9) and the ~10 kDa lower protein variant in case 10 (consistent with the loss of exon 4). Red asterisks mark specific signals in the ACG1A cases (consistent with biallelic stop mutations and a predicted protein size of about 142 kDa in case A2 and 134 kDa in case A1). Additional bands for GMAP-210 marked with blue asterisks in CTL cells may represent shorter variants or proteolytic cleavage products. (**E**) Green asterisks mark the putative GMAP-190 protein corresponding to *TRIP11*-ΔEx9short.

All disease-associated mutations in *TRIP11* are predicted to alter the primary structure of the GMAP-210 protein (Figure 4A) and caused a strong reduction of GMAP-210 abundance in patient-derived primary cells (Figure 4B). However, longer exposure revealed weak signals at expected sizes for the respective variants in all ODCD cases, whereas GMAP-specific bands remained at or below the detection limit in ACG1A, indicating near-complete loss of the protein (Figure 4, B–E).

Case 10 carries the most prevalent splice mutation affecting exon 4 and a null allele (Table 2 and Table 3). We expected a GMAP protein of approximately 10 kDa lower molecular weight, since exon 4 codes for 92 amino acids of the coiled coil sequence (Figure 4A). Accordingly, a single lower band was found in patient-derived fibroblasts using antibodies directed against the amino-terminus of GMAP-210 (Figure 4, B and D). The identified GMAP protein corresponds to *TRIP11*-ΔEx4 and is identical to a GMAP-200 isoform previously characterized in HeLa cells (19). Quantitative analysis indicated that GMAP-200 protein levels in case 10 only amount to approximately 30% compared with full-length GMAP-210 in WT controls (Figure 4C).

In case 3, we expected a GMAP protein missing the 29 amino acids encoded by exon 9 (Table 2, Table 3, and Figure 4A). Accordingly, Western blot demonstrated a 3-kDa lower GMAP-207 protein in fibroblasts corresponding to *TRIP11*-ΔEx9 (Figure 4, B, D, and E) bearing the exon 9 missplice and a compound *TRIP11* nonsense mutation (Table 2 and Table 3). Using amino- and carboxy-terminal antibodies, only a faint single band was visible with an abundance of less than 10% as compared with the mean signal of full-length protein in healthy controls (Figure 4C). As the corresponding *TRIP11*-ΔEx9 mRNA was not decreased below the level of haplo-insufficiency (Figure 2C), we concluded that the GMAP-207 protein variant was either less efficiently translated or less stable than the full-length GMAP-210.

In case 6, bearing the c.5416A>G splice/missense mutation and an early truncating mutation on the other allele predicting NMD (p.Lys541Argfs*17), we expected a full-length missense GMAP-210 protein of low abundance, as more than half of the remaining *TRIP11* transcripts encoded the p.Met1806Val amino acid substitution (Figure 4A, Supplemental Figure 3B) and the concentration of total *TRIP11* mRNA was strongly reduced (Figure 2C). Accordingly, we only detected trace amounts of a protein with the same electrophoretic mobility as WT GMAP-210 (Figure 4, B, D, and E). Thus, endogenous and disease-associated splice variants of *TRIP11* were translated in all ODCD cases analyzed. To determine if the resulting marginal amounts of shorter GMAP proteins were sufficient to rescue the cellular consequences of GMAP-210 deficiency (1, 20, 26), we characterized the residual protein function in detail.

### Residual GMAP proteins are correctly anchored, but the Golgi structure is altered in ODCD.

**Figure 5:**
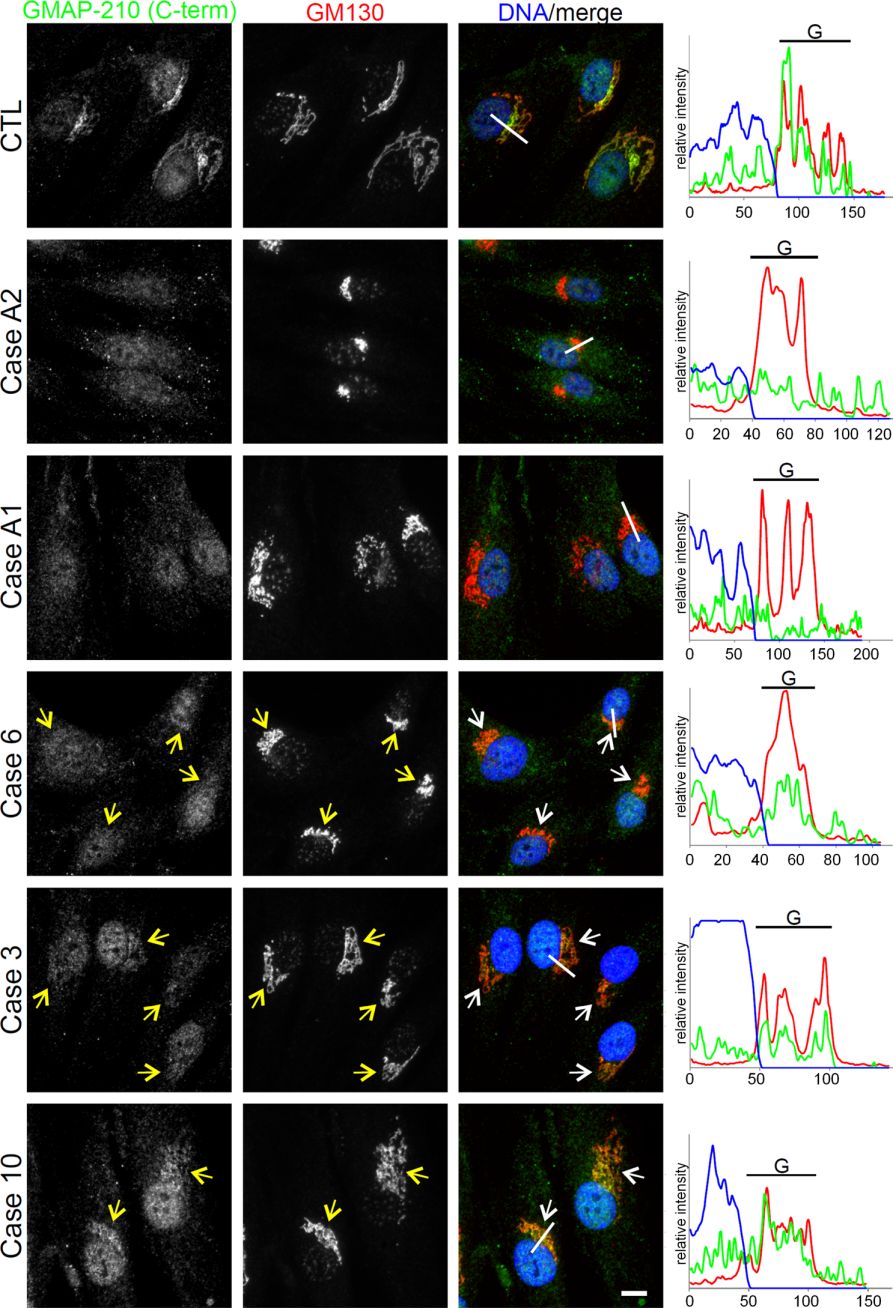
Residual GMAP protein variants are found at the *cis*-Golgi in odontochondrodysplasia (ODCD). Wide-field microscopy of control and patient-derived fibroblasts costained with carboxy-terminal GMAP-210 antibody and the *cis*-Golgi marker GM130. Yellow and white arrows mark the *cis*-Golgi. Nuclear DNA was stained with Hoechst 33342 dye. Note that the GMAP-210 carboxy-terminal antibody gives a nuclear staining, in addition to detecting Golgi-associated GMAPs. The area selected for red-green-blue (RGB) profiling is indicated by a white line. Fluorescence intensity is shown in RGB profile plots (right). G, Golgi apparatus. Scale bar: 10 μm.

Recent studies have shown that GMAP-210 acts as a tether for transport vesicles at the *cis*-Golgi in human cells (7, 8, 20). Therefore, we investigated the subcellular distribution and local abundance of disease-associated GMAP variants in patient-derived primary cells. Immunofluorescence microscopy revealed a strong signal for GMAP-210 at the Golgi apparatus in controls, costaining with the *cis*-Golgi marker GM130 (Figure 5). In contrast, no GMAP-210–specific signal was detected in fibroblasts of ACG1A cases A1 and A2 bearing biallelic nonsense mutations in *TRIP11*. Remarkably, while GMAP-210 staining was less intense in ODCD than in controls, red-green-blue (RGB) profile blots demonstrated that the residual protein was still correctly targeted to the *cis*-Golgi in each case (Figure 5). We further noted that there was compaction of the Golgi apparatus in ACG1A and in ODCD case 6, which had the lowest amount of residual GMAP protein. In contrast, a close-to-normal Golgi ribbon morphology was seen in case 10, where the strongest GMAP-specific signals were detected. These findings were verified using Golgi subcompartment–specific antibodies (Supplemental Figure 6).

To confirm their ability to anchor to the *cis*-Golgi, GMAP variants lacking exons 4 and 9 were analyzed when overexpressed at higher levels in generic cell lines (Supplemental Figure 7A). No alteration was seen in subcellular localization of overexpressed GMAP-200 (encoded by *TRIP11*-ΔEx4) and GMAP-207 (encoded by *TRIP11*-ΔEx9); both proteins were anchored at the *cis*-Golgi as efficiently as full-length protein, which is via the conserved carboxy-terminal GRIP-related Arf-binding (GRAB) domain (Supplemental Figure 7B) (27). Therefore, neither the amino-terminus encoded by the exons 1–5, nor the domains encoded by exons 4 and 9 alone, were critical for the Golgi anchoring of GMAP-210, which was expected from previous studies (27, 28).

**Figure 6:**
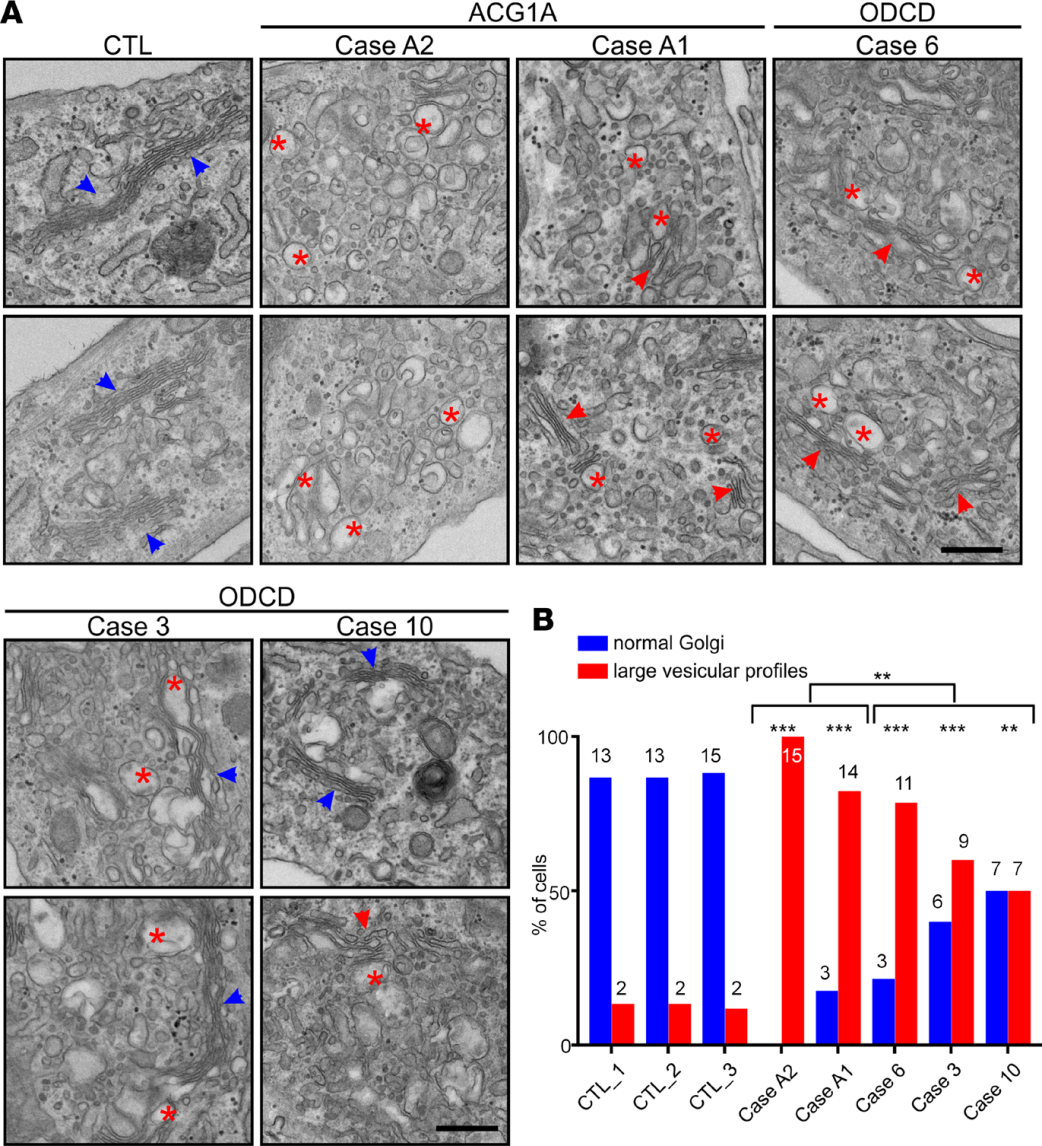
Golgi ultrastructure in achondrogenesis 1A (ACG1A) and odontochondrodysplasia (ODCD). (**A**) Transmission electron microscopy (TEM) of patient and control cells. Blue arrowheads mark normal Golgi stacks; red arrowheads mark Golgi mini stacks, while red asterisks depict Golgi vesiculation. Scale bar: 500 nm. (**B**) Quantification of cells with abnormal vesiculation, defined as peri-Golgi circular profiles with a diameter of >100 nm (*n* > 25). The number of cells with large vesicular profiles was compared using a χ^2^ test. Statistical significance cut-offs were set as follows: **P < 0.01 and ***P < 0.001.

Transmission electron microscopy (TEM) corroborated the critical structural function of GMAP-210 for the human Golgi apparatus, since the cisternal stack architecture was completely lost in ACG1A (29). Numerous large vesicular profiles accumulated in the Golgi region, and none of the analyzed GMAP-210–negative cells showed distinguishable Golgi ribbons (Figure 6A). In accordance with the immunofluorescence data (Figure 5 and Supplemental Figure 6), ultrastructural abnormalities were considerably milder in ODCD. However, Golgi stacks in ODCD exhibited dilated cisternae, with accumulation of small and large vesicular profiles (Figure 6A), likely corresponding to cisternal remnants and/or untethered transport vesicles (20). Quantification of the ultrastructural disease phenotype revealed that Golgi vesiculation correlated negatively with the amount of GMAP proteins left in patients’ cells (Figure 6B). ER ultrastructure was unchanged in ACG1A and ODCD fibroblasts (Supplemental Figure 8), contrary to observations in *Trip11*-mutant mouse chondrocytes (1).

To assess the functional competence of the individual GMAP variants, they were overexpressed in cells derived from an ACG1A case. ODCD-associated variants lacking exons 4 and 9, as expressed in cases 10 and 3, restored normal Golgi organization in GMAP-210–deficient cells (Supplemental Figure 9). The GMAP-190 variant lacking the amino-terminal tethering motifs was reduced in its ability to rescue, consistent with the notion that the function of the full-length GMAP-210 protein as a vesicle tether is also important for Golgi organization. Putting results together, we reasoned that GMAP-210 is a nonredundant structural component of the Golgi apparatus in humans, and even minimal quantities of ODCD-associated GMAP variants are sufficient to rescue structural abnormalities. The phenotype of ODCD, therefore, appears primarily due to reduced abundance of the GMAP-210 protein.

### An IFT20 Golgi pool is maintained by GMAP variants in ODCD.

In addition to acting as a vesicle tether, GMAP-210 physically interacts with IFT20, which has recently been described as an essential regulator of normal skeletal development (5, 30). Furthermore, IFT20 is dispersed and degraded when GMAP-210 is absent (5). In ciliated cells, IFT20 is a component of the intraflagellar transport complex B (IFT-B) and the only known adapter mediating protein export from the Golgi apparatus to the primary cilium (5). However, costaining with acetylated tubulin revealed normal cilia and no difference in the ciliary signal of IFT20 in serum-deprived fibroblast from controls, ACG1A, and ODCD (Supplemental Figure 10 and Supplemental Figure 11).

**Figure 7:**
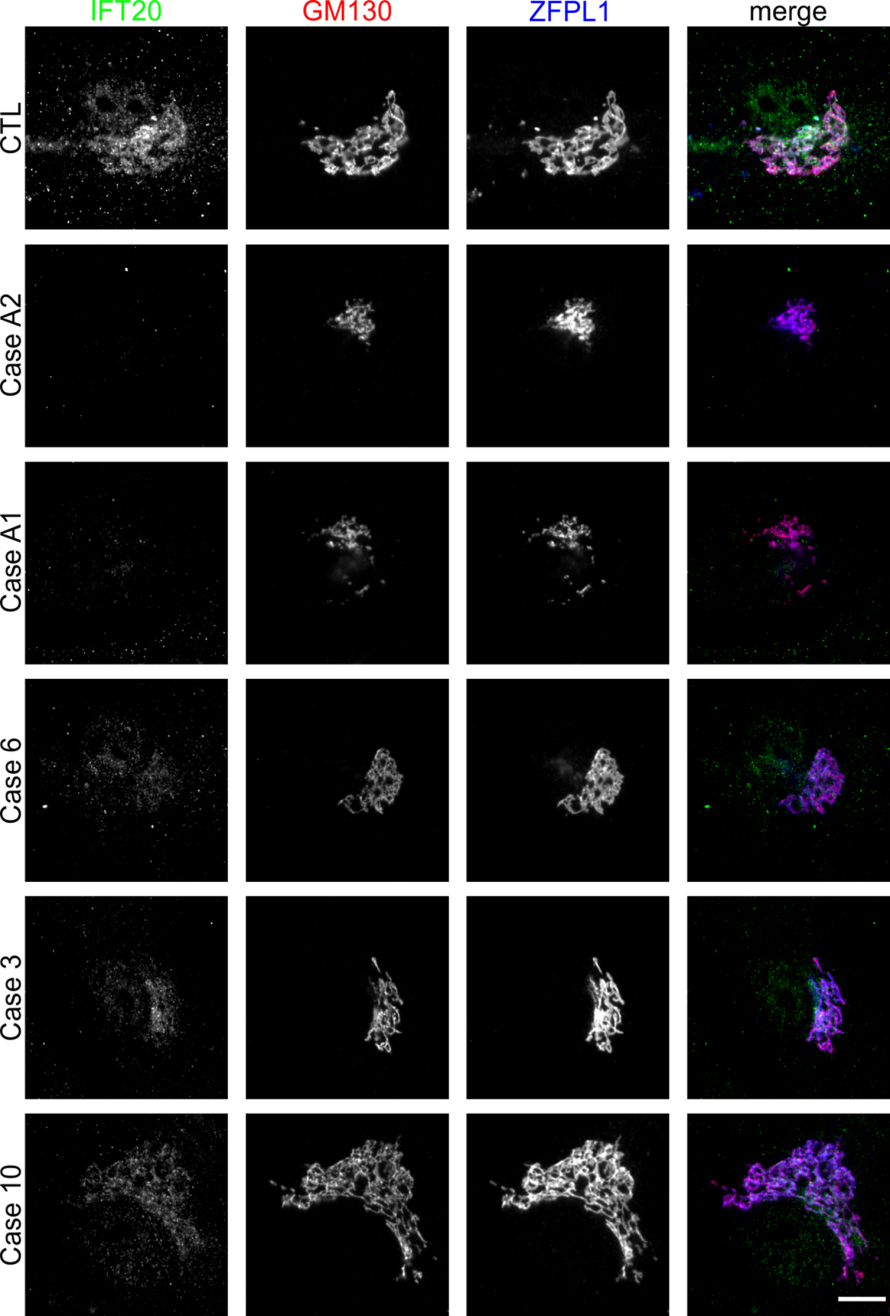
Golgi anchoring of IFT20 in odontochondrodysplasia (ODCD). Wide-field microscopy of control and patient fibroblasts costained with antibodies directed against the *cis*-Golgi proteins GM130 and ZFPL1, as well as antibodies directed against IFT20. Scale bar: 10 μm.

A recent report has described that IFT20 also regulates the nucleation of Golgi-derived microtubules, thereby promoting ribbon formation and polarized secretion in human chondrosarcoma cells (31). Therefore, we next focused on possible nonciliary roles of IFT20 and found no protein at the Golgi apparatus in GMAP-210–deficient ACG1A cells using immunofluorescence microscopy (Figure 7). In contrast, residual Golgi staining was detected in all ODCD cases, indicating that trace amounts of GMAP protein variants are sufficient to target some IFT20 to the Golgi apparatus, with the amount correlating to the amount of GMAP present in each case (Figure 7). Both exogenous full-length GMAP-210 and shorter GMAP variants lacking exons 4 or 9 restored IFT20 Golgi anchoring when overexpressed in ACG1A patient–derived cells (Supplemental Figure 12), consistent with the IFT20 binding site lying outside of these regions of the protein (Figure 4A) (5).

The ability of individual GMAP splice variants to bind to IFT20 in cells was further tested by inducibly targeting the variants to mitochondria (32) and assessing the binding of IFT20 to relocated GMAP by immunofluorescence microscopy (Supplemental Figure 13A) (8). As shown in Supplemental Figure 13B, IFT20 was cotranslocated to mitochondria by GMAP-207 (encoded by *TRIP11*-ΔEx9), GMAP-200 (encoded by *TRIP11*-ΔEx4), and GMAP-190 (encoded by *TRIP11*-ΔEx9short), demonstrating that they all contain a functional IFT20 binding site (5). We propose that complete loss of IFT20 from the Golgi apparatus in ACG1A contributes to the more severe cellular phenotype compared with ODCD, where residual IFT20 is present at the Golgi.

### ODCD-associated GMAP variants sustain bulk secretory traffic in fibroblasts but are defective in glycan processing at the Golgi.

It has been shown that GMAP-210 selectively interacts with transport vesicles via its ALPS motif and a second amino-terminal tethering motif (21, 33). Subsequent interaction of RAB2 mediates vesicle processing at the *cis*-Golgi in human cells (20). Using the mitochondrial rerouting assay, we analyzed vesicle capture by the GMAP variants in vivo (32). As observed previously for the full-length protein (8, 20), the Golgi-resident enzyme GalNAc-T2 accumulated at GMAP-positive mitochondria in the case of the ODCD variants, indicating capture of Golgi-derived vesicles (Supplemental Figure 14A). The only variant unable to capture GalNAc-T2–containing vesicles was GMAP-190 (encoded by *TRIP11*-ΔEx9short) because of its missing amino-terminal tethering motifs. Thus, ODCD-associated GMAP splice variants are competent to tether transport vesicles to the *cis*-Golgi, as well to bind IFT20 (Supplemental Figure 14B). This again supports the notion that the ODCD phenotype arises from reduced protein abundance, as opposed to any inherent loss of functionality of variant GMAP proteins.

**Figure 8:**
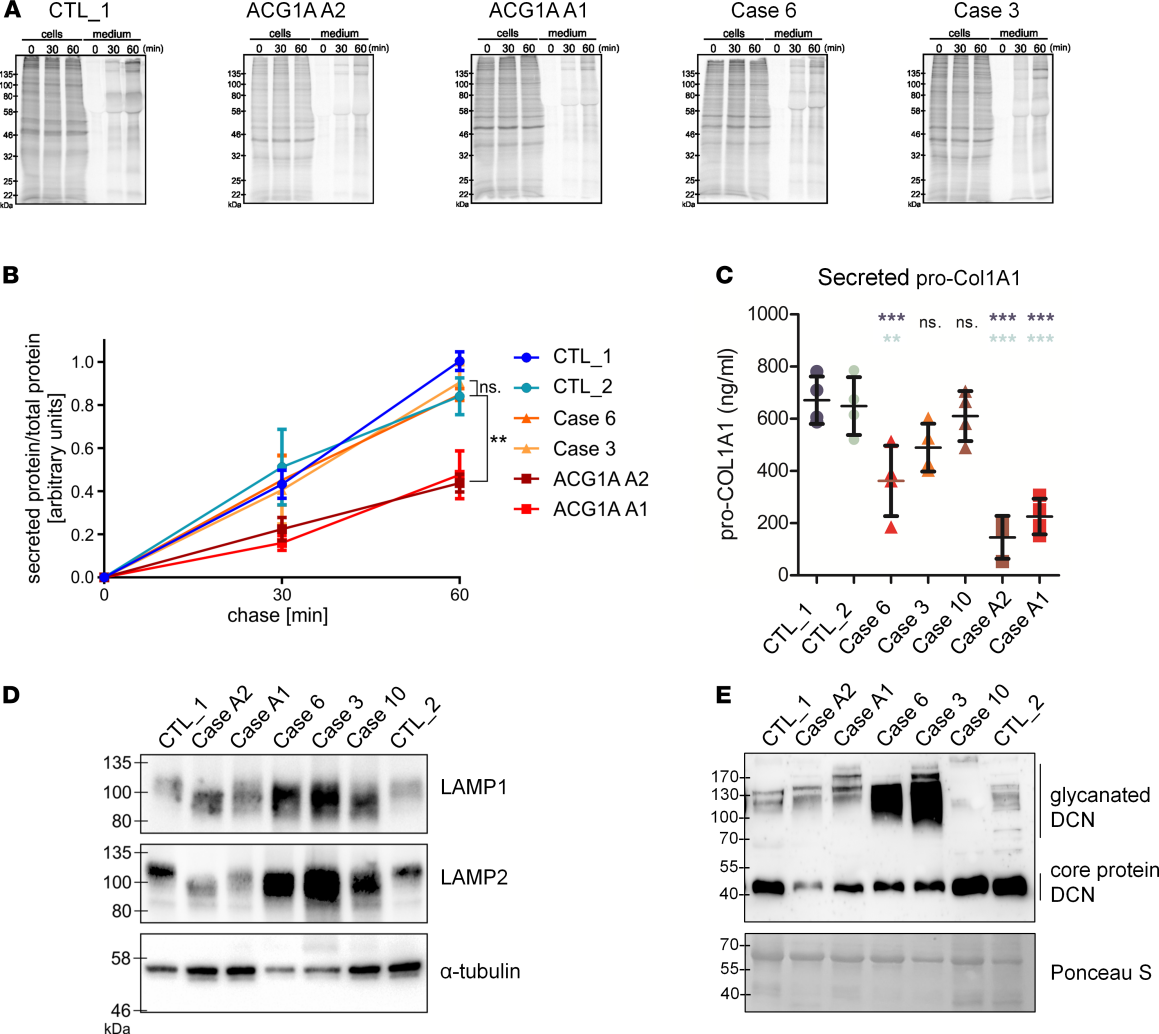
Normal global secretory traffic but defective glycan processing by the Golgi apparatus in odontochondrodysplasia (ODCD). (**A**) Pulse-chase analysis of protein secretion of control and patient fibroblasts. Cells were starved for 1 hour in methionine- and cysteine-free medium and pulsed for 20 minutes with fresh starvation medium containing 50 μCi/ml [^35^S]methionine and [^35^S]cysteine protein labeling mix. Cells were chased in medium containing unlabeled methionine and cysteine for 0, 30, or 60 minutes and lysed. Radiolabeled cellular (cells) and secreted (medium) proteins, which were precipitated by trichloroacetic acid, were subjected to sodium dodecyl sulfate polyacrylamide gel electrophoresis and visualized by phosphor-imaging. (**B**) Ratio of secreted/total proteins at the indicated time points. Data represent mean ± SEM from 4 independent experiments (1-way ANOVA with Dunnett’s multiple comparisons test); **P < 0.01. (**C**) Pro-collagen, type 1, α-1 (pro-COL1A) concentration in conditioned media of primary fibroblasts determined by enzyme-linked immunosorbent assay. Data represent mean ± SD of quadruplicates (1-way ANOVA with Dunnett’s multiple comparisons test); **P < 0.01, ***P < 0.001. (**D**) Western blot analyses of LAMP1 and LAMP2 protein species in whole-cell lysates of patient and control fibroblasts. Hypo-glycosylated LAMP1 and LAMP2 proteins in achondrogenesis 1A (ACG1A) and ODCD run at a lower molecular weight range than in controls. (**E**) Western blot of secreted decorin (DCN) in conditioned supernatants of primary fibroblasts. High-molecular weight species, corresponding to glycanated DCN, include both slower and faster migrating forms in ACG1A and ODCD; different abundance of the core protein indicates a secretory defect. Ponceau S staining of the membrane was used to assess the relative amount of precipitated and transferred total protein.

Since vesicle tethering is required for vesicular transport in the cell, we wondered whether the reduced abundance of GMAP proteins in ODCD would impact on secretory trafficking, all the more so as a reduced capacity to locate IFT20 at the Golgi may also have an influence on Golgi function (30, 31). Therefore, total protein secretion by primary fibroblasts was measured as described (Figure 8A) (34). The cellular capacity to export nascent proteins into extracellular space correlated with the amount of GMAP present at the Golgi. Compared with WT controls, the secretory output of ACG1A fibroblasts was reduced to approximately 50% at all time points (Figure 8B). In contrast, bulk secretion was normal in ODCD fibroblasts, which contain residual amounts of GMAP variants (Figure 8, A and B).

However, the remaining GMAP function in ODCD was not sufficient to ensure normal transport of all cargoes, as the levels of procollagen, type 1, α-1 (pro-COL1A1) secreted by patient-derived fibroblasts were reduced compared with controls but were still significantly higher than in ACG1A (Figure 8C). In accordance with residual golgin function in ODCD, pro-COL1A1 secretion correlated positively with the amount of GMAP/IFT20 complex at the Golgi; this may also endorse a role of IFT20 for the trafficking of specific substrates (Figure 8C) (30, 31).

To further assess Golgi functionality in the patient fibroblasts, glycosylation of the lysosomal membrane proteins LAMP1 and LAMP2 was analyzed. Their glycosylation is dependent upon efficient intra-Golgi trafficking of processing enzymes and serves as a sensitive readout of Golgi functionality (35). As shown in Figure 8D, there was impairment of LAMP1 and LAMP2 glycosylation in all patient-derived fibroblasts, as indicated by their lower apparent molecular weight upon SDS-PAGE. A severe glycosylation defect was also observed for secreted decorin, which serves as a protein backbone for chondroitin and dermatan sulfate chains in the extracellular matrix (ECM). Decorin has diverse functions that depend on the length of the attached glycosaminoglycan (GAG) chains, including the assembly of collagen type I fibrils and the release of cytokines (36, 37). Similar to aberrant LAMP protein mobility, the pattern of high-molecular weight species representing glycanated decorin differed greatly between controls and patients. However, these changes were more variable, with both larger and smaller glycanated forms detected in *TRIP11*-mutant cells (Figure 8E). Furthermore, the ratio of glycanated decorin to core protein at approximately 40 kDa was different from controls but not consistently changed in patients, with particular variances in the abundance of high-mobility forms among ODCD cases (Figure 8E). This is also the result of reduced nonglycanated core protein at 40 kDa in ACG1A and severe ODCD, matching the findings for secreted pro-COL1A1 (Figure 8D) and supporting the notion that impaired protein trafficking of specific substrates (6) and their altered posttranslational modification may contribute independently to the pathogenesis and severity of *TRIP11*-related disorders. We therefore conclude that, in both ACG1A and ODCD, Golgi functionality is severely impaired and disturbs secretion and posttranslational processing of specific ECM components, but only in ACG1A is impairment strong enough to manifest as reduced secretory traffic of global cargo.

### Bulk ECM secretion is disrupted in TRIP11-mutant chondrogenic cells.

**Figure 9:**
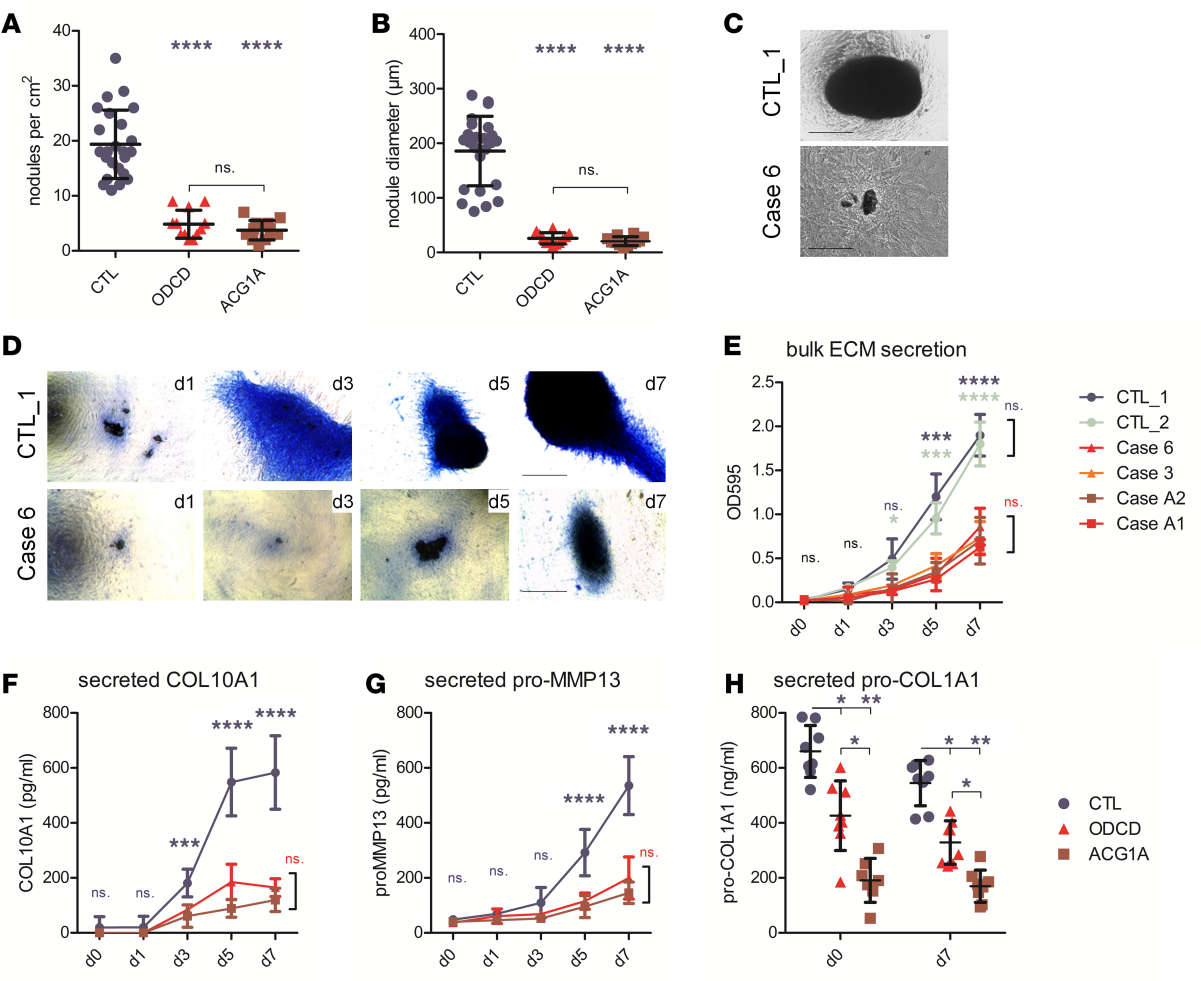
Impaired synthesis of extracellular matrix (ECM) components and disrupted chondrogenic differentiation of *TRIP11*-mutant cells. (**A**) Number of chondrogenic nodules at day 7 of transdifferentiation of fibroblasts (fibroblast-induced chondrocytes, FDC) in controls and patients. (**B**) Size of the chondrogenic nodules at day 7, as exemplified for case 6 by inverse microscopy of live cells in (**C**). Scale bar: 100 μm. (**D**) Alcian blue staining of transdifferentiated patient-derived primary and matched control FDC cultures at days 1–7 of chondrogenic differentiation demonstrates reduced matrix production in patient cells. Scale bars: 150 μm. (**E**) Quantification of total glycosaminoglycan/proteoglycan content in FDCs; data points represent the mean of triplicate values (*n* = 3); error bars indicate ± SD. (**F** and **G**) Quantitative ELISA shows that secreted markers of terminal differentiation, COL10A1 and pro-MMP13, are significantly reduced in supernatants of patient-derived cultures at late time points of the transdifferentiation protocol. CTL, controls 1 and 2; ODCD, odontochondrodysplasia cases 3, 6, and 10; ACG1A, achondrogenesis 1A cases A1 and A2. Data points represent the mean of quadruplicate values (*n* = 4); error bars indicate ± SD. (**H**) Quantitative ELISA of pro-collagen, type 1, α-1 (pro-COL1A1) in conditioned FDC supernatants. Data represent mean ± SD of quadruplicates (2-way ANOVA with Bonferroni’s post hoc test); *P ≤ 0.05, **P < 0.01, ***P < 0.001, ****P < 0.0001.

To investigate the impact of *TRIP11* mutations on human skeletal development, we then transdifferentiated primary dermal fibroblasts into mature chondrocytes (24). When cultured on an aggrecan-coated surface, fibroblasts can be induced by conditioned media to undergo a continuous differentiation process toward a chondrocyte lineage (FDC) (24, 38). The chondrocyte differentiation state at different days corresponds to that of the distinct histological zones in the growth plate (39). Proliferating FDCs form 3-dimensional chondrogenic nodules consisting of secretory cells surrounded by cartilage matrix, which grow up to a size of several hundred micrometers (Figure 9, A–C) and can be specifically stained by the cartilage proteoglycan dye Alcian blue (Figure 9D) (40). Total ECM synthesis, as quantified by Alcian blue extraction of FDC cultures, increases with progressing FDC differentiation stage and peaks at day 7 in WT cells (Figure 9E). In *TRIP11*-mutant cells, the total number and the mean size of chondrogenic nodules were significantly reduced (Figure 9, A and B), indicating that the production of bulk glycosylated proteoglycans by FDCs was disrupted. Unexpectedly, no significant difference was observed between ACG1A and ODCD (Figure 9E), in contrast to a normal global secretory capacity of the latter in fibroblasts (Figure 8B). Based on conditional *Trip11*-KO mice, it was recently proposed that GMAP-210 deficiency in chondrocytes only impairs the secretion of selected ECM components that are retained intracellularly (6). Our results indicate a profound secretory defect of *TRIP11*-mutant chondrogenic cells for ECM proteoglycans, which is not improved by the hypomorphic GMAP variants in ODCD. We therefore questioned whether the severe impact of GMAP-210 dysfunction on human chondrocytes was directly due to a rate-limiting trafficking defect of highly secretory cells (1, 6).

### Hypertrophic chondrocyte differentiation is impaired in ACG1A and ODCD.

In the few published histological studies (3, 41, 42), growth plates of ACG1A fetuses show a reduction of total epiphyseal cartilage and the absence of columnar chondrocytes in the prehypertrophic and hypertrophic zones (42). Accordingly, collagen, type X, α-1 (COL10A1), a specific cargo secreted by terminal hypertrophic chondrocytes at high levels, was not detectable in the ECM (42). In line with published data, no columnar chondrocytes were found in the femoral growth plate of case A5 (Supplemental Figure 15A). However, overall cell numbers were not reduced as the reserve and proliferative zones were expanded and hypercellularity was observed (Supplemental Figure 15, A and B).

**Figure 10:**
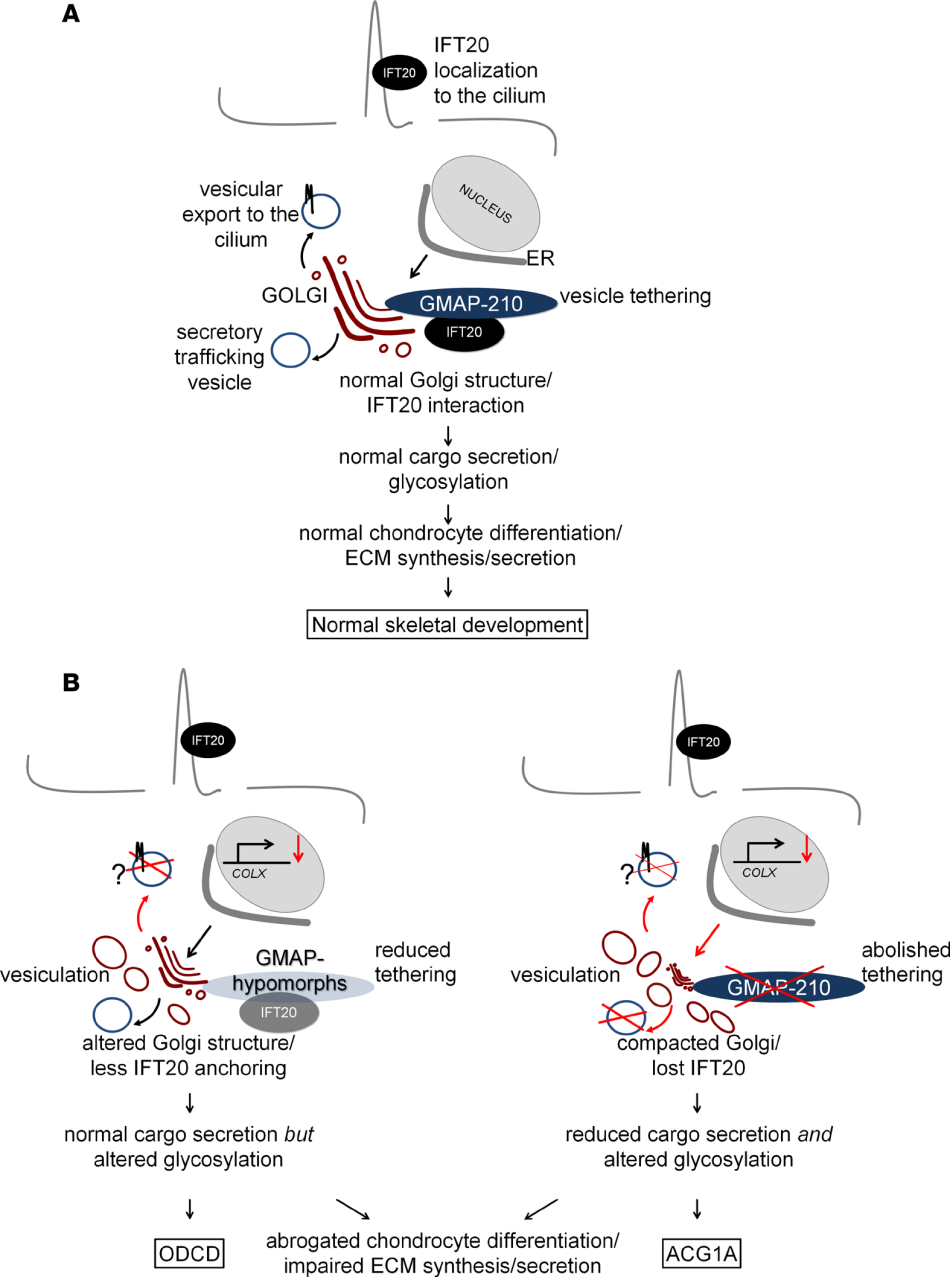
The function of the GMAP/IFT20 complex in development and disease. (**A**) Functioning as a membrane tether, GMAP-210 anchors IFT20 to the *cis*-Golgi apparatus. IFT20 is involved in cargo sorting to the cilium (e.g., polycystin-2). Golgi organization is essential for proper secretory transport and posttranslational protein modification. (**B**) Odontochondrodysplasia-associated (ODCD-associated) GMAP hypomorphs and natural splice variants partially maintain the structure of the Golgi apparatus. In achondrogenesis 1A (ACG1A), GMAPs are fully lost. Golgi structure is disrupted, and bulk secretory traffic is reduced. Aberrant posttranslational protein modification disrupts chondrocyte differentiation in both ODCD and ACG1A. Impaired synthesis and secretion of extracellular matrix (ECM) proteoglycans cause skeletal disease.

Addressing the possibility that reduced ECM synthesis by *TRIP11*-mutant human FDCs was nonetheless due to lower cell numbers, we found that proliferation was not impaired during chondrogenesis (Supplemental Figure 15C). Moreover, cell viability remained in the same range at all stages of induced differentiation. Contrary to previous observations in constitutive *Trip11*-mutant mice (1), no evidence was found that human GMAP-210 deficiency causes ER swelling (Supplemental Figure 8) and triggers apoptosis (Supplemental Figure 15D). We therefore hypothesized that GMAP dysfunction disrupts endochondral ossification, and we studied chondrocyte differentiation markers in FDCs. Late-stage FDCs display the properties of prehypertrophic and terminal hypertrophic chondrocytes, characterized by a strong upregulation of matrix metalloprotease 13 (MMP13) and COL10A1 (Figure 9, F and G) at days 5–7. In both ACG1A- and ODCD-derived cells, the terminal hypertrophic differentiation state was never attained, as indicated by persistently low levels of *COL10A1* mRNA and protein (Figures 9F and Supplemental Figure 15E). Furthermore, whereas pro-MMP13 was induced in late-stage control FDCs, levels in *TRIP11*-mutant cells remained as low as in nondifferentiated fibroblasts (Figure 9G and Supplemental Figure 15F). Conversely, analysis of pro-COL1A1 in mature chondrogenic cells revealed that the secretory trafficking of some cargoes was still significantly more efficient in ODCD than in ACG1A (Figure 9H). *COL1A1* is ubiquitously expressed and not restricted to hypertrophic chondrocytes as the other 2 studied markers. We conclude that low levels of COL10A1 and pro-MMP13 in ODCD do not solely reflect a rate-limiting trafficking defect of chondrocytes, but are also the result of failed induction of terminal chondrogenic differentiation. These findings support the notion that GMAP-210 is a critical regulator of hypertrophic chondrocyte differentiation and that mutations in *TRIP11* cause a common chondrocyte maturation defect in ACG1A and ODCD (Figure 10).

## Discussion

By follow-up of published and additional patients, we studied the natural history in the largest series of ODCD cases to date, to our knowledge. Clinical-radiographic overlap with ACG1A and a recessive inheritance pattern were instrumental for the identification of *TRIP11* as the causative gene in ODCD. Our clinical observations add 2 important aspects to the description of ODCD (10–12): first, there is considerable clinical variability in *TRIP11*-related disorders, with early lethality and milder postnatal phenotypes occurring within the same family, and second, there are extraskeletal disease manifestations in ODCD that implicate GMAP-210 in human renal and cerebral development (15). The role of GMAP-210 in organogenesis and tissue homeostasis may, thus, be wider than assumed on the basis of current *Trip11*-KO models. These models all bear the disadvantage of perinatal lethality, precluding the recognition and analysis of later-onset phenotypes (1, 5, 6). Comprehensive characterization of individual mutations revealed that all ODCD patients, in addition to a *TRIP11*-null allele, carry compound heterozygous splice variants, which are translated into low-abundance GMAP proteins. Our findings establish that ODCD is genetically homogeneous and that hypomorphic mutations of *TRIP11* are the genetic basis for a range of ODCD phenotypes.

Is there hence a consistent genotype-phenotype correlation in *TRIP11*-related disorders? Our results support that a complete loss of *TRIP11* fatally disrupts skeletal development and invariably causes early embryonic lethality in ACG1A, whereas recurrent splice mutations are associated with a milder but variable spectrum of postnatal clinical phenotypes. At the cellular level, phenotypic severity is clearly correlated with a reduction of functional GMAP proteins at the Golgi apparatus. However, the very same hypomorphic mutations may lead to both early lethal disease and milder ODCD. A radiographic diagnosis of ACG1A and ODCD in different children originating from the same parents may be primarily due to variable penetrance of *TRIP11* missplicing between individuals, resulting in constitutively different amounts of residual GMAP protein. Thus, our results make the prediction of clinical outcome solely based on genotype difficult in genetic counselling and clinical care. Although our functional data corroborate that ODCD is the milder allelic form of ACG1A, clinical variability associated with hypomorphic *TRIP11* mutations is too high for a reliable genotype-phenotype correlation, at present.

How do hypomorphic *TRIP11* alleles rescue the embryonic lethality? Notably, a drastic reduction of full-length GMAP-210 protein was evident in both ACG1A and ODCD, leaving only trace amounts of altered GMAP proteins in the latter; a milder clinical phenotype may, thus, still have extrinsic reasons, as well. However, by comparing the cellular phenotypes of ACG1A and ODCD in primary cells, we identified surprisingly clear-cut differences that were directly attributable to preserved golgin functions of residual GMAP proteins: first, ODCD-associated splice mutations were permissive for the generation of low-abundant proteins, while all mRNA isoforms of *TRIP11* were effectively eliminated by NMD in ACG1A. Golgi morphology and ultrastructure was much less disordered in ODCD than in ACG1A, where the organelle was compacted, fragmented, and dispersed into irregular vesicular profiles. Second, only in ODCD, there was a sustained recruitment of IFT20 to the Golgi apparatus, which again was directly dependent on residual GMAP quantity in patient cells. All ODCD-associated and natural GMAP splice variants retained an IFT20 binding site and rescued the dispersal of the IFT20 Golgi pool in GMAP-210–negative cells. IFT20 may directly be involved in the ER-to-Golgi transport of specific cargoes (30). Recent data indicates that IFT20 regulates the nucleation of Golgi-derived microtubules in nonciliated cells by physical interaction with the GM130–A-kinase anchor protein 450 (GM130-AKAP450) complex (31). The formation of a microtubule network emanating from the *cis*-Golgi membrane by IFT20 was shown to promote ribbon formation and to increase the efficiency of anterograde transport through the Golgi complex (31). Sustained Golgi targeting of IFT20 in ODCD may, therefore, attenuate the structural and functional Golgi defect caused by GMAP-210 deficiency. The importance of a preserved GMAP/IFT20 complex for a milder cellular and clinical phenotype of ODCD was strongly supported by a third essential difference to ACG1A detected in patient-derived cells: whereas a strong reduction of total membrane trafficking was observed in ACG1A, the global secretory capacity of the Golgi was normal in ODCD, at least in primary fibroblasts.

Taken together, minimal amounts of functional GMAP are sufficient to restore Golgi function to a degree that permits pre- and postnatal survival in ODCD. Rescue of the structural and functional Golgi defects likely depends on vesicle tethering but may also depend on the recruitment of IFT20 by GMAP to the *cis*-Golgi. In turn, complete loss of the GMAP-210/IFT20 complex in ACG1A leads to early embryonic lethality due to a global trafficking defect of the Golgi apparatus (Figure 10).

How do developmental anomalies emerge in *TRIP11*-related disorders? Our clinical and functional data suggest that independent mechanisms may contribute to organ-specific disease manifestations in ACG1A and ODCD: in the skeleton, the structural and functional defects of the Golgi apparatus disrupt glycan processing with a pronounced and early impact on chondrocyte differentiation. A block in cellular differentiation impairs the evolution of hypertrophic chondrocytes, which synthesize key cartilage matrix proteins in the growth plate, including COL10A1. A more severe skeletal phenotype in ACG1A may be due to the global reduction of secretory traffic. The etiology of the differentiation defect remains currently unclear, but we hypothesize that aberrant posttranslational processing and secretion of a limited number of secreted developmental regulators with key importance for early chondrogenesis, like decorin (36, 37), are major mechanism in the skeleton (43). This view is supported by the fact that aberrant posttranslational processing of selected glycans causes ACG1B, a lethal skeletal dysplasia with a highly similar skeletal phenotype to ACG1A (44).

Our studies did not provide support for a cilium-dependent pathogenesis in *TRIP11*-related disorders, as cilia in GMAP-210–deficient fibroblasts had normal length and exhibited no structural abnormalities. Furthermore, ciliary targeting of IFT20 itself was normal in ODCD and ACG1A, in spite of its partial, or complete, dispersal from the Golgi. However, known substrates of GMAP-210/IFT20-mediated ciliary export, like polystcystin-2 (5) and fibrocystin (45), are not expressed in fibroblasts or chondrocytes, so we were unable to assess a potential functional defect of primary cilia in *TRIP11*-mutant human cells. Therefore, we cannot exclude that a ciliary export defect of other currently unknown proteins contributes to the developmental phenotype.

None withstanding, radiographic features of ACG1A and ODCD are characteristic of skeletal ciliopathies — in particular, the combination of thoracic dystrophy, brachydactyly with cone-shaped epiphyses, and a trident pelvis (14). The pattern of extraskeletal disease manifestations in ODCD with variable dental, renal, and cerebral anomalies is also suggestive of an underlying cilium-based pathogenesis. The same organs are affected in an overlapping condition known as cranioectodermal dysplasia, or Sensenbrenner syndrome, which is caused by hypomorphic mutations of *IFT122*, another component of the IFT-B complex (46). Impaired release of IFT20 from the Golgi and subsequent disturbance of ciliary export was recently implicated as an underlying disease mechanism in another ciliopathy with skeletal, renal, and retinal involvement (47). It is therefore tempting to speculate that at least the late-onset cystic renal degeneration observed in ODCD may be primarily due to impaired ciliary transport functions of IFT20, as the conditional deletion of *Ift20* during renal development causes polycystic kidney disease and aberrant WNT signaling in neonatal mice (48). A similar significance of IFT20 for the other extraskeletal manifestations in ODCD remains to be investigated. We propose that a genuine disease model of ODCD will be a straightforward way to unravel the organ-specific tasks of GMAP-210 in development.

In conclusion, we demonstrate here that biallelic mutations of *TRIP11* cause a spectrum of skeletal phenotypes whose severity is primarily based on impaired secretory trafficking and aberrant glycan processing by the Golgi apparatus. Our results support a critical nonciliary role of IFT20 for Golgi organization and membrane trafficking in the growth plate. Further elucidation of the skeletal and extraskeletal disease manifestations of *TRIP11*-related disorders will allow a dissection of the multiple functions of the GMAP-210/IFT20 complex in vesicular transport, protein modification, and ciliary transport.

## Methods

### Patients and mutation analysis.

DNA was obtained by standard extraction procedures (Qiagen). For genomic analysis and mutation detection, exons including intron-exon boundaries of *TRIP11* were amplified by PCR using standard protocols as described (29), primers are listed in Supplemental Table 2. Array CGH analysis was carried out using 180K oligonucleotide arrays, as described previously, on an Agilent platform (49). Image quantification, array quality control, and aberration detection were performed by DNA analytics software packages (CytoGenomics) according to the manufacturer’s instructions (Agilent).

### Cell culture, treatment, and transfection.

COS7 and HeLaM (ATCC) cells were grown at 37°C and 5% CO_2_ in DMEM supplemented with 10% (vol/vol) FBS, 1 mM L-glutamine, 1% nonessential amino acids (NEAA), and 1% penicillin-streptomycin mix (all from Invitrogen). Human dermal fibroblasts from patients and healthy controls were obtained from skin biopsies and expanded to passage 3 (p3). Fibroblasts were maintained in 90% DMEM (4.5 g/l glucose, Invitrogen), 10% FBS (from PAA Laboratories), 0.1 mM NEAA, 100 U/ml penicillin, and 100 μg/ml streptomycin (all from Invitrogen) in humidified atmosphere, 37°C, and 5% CO_2_. Monocilia were induced in subconfluent cultures seeded on glass coverslips by serum starvation for 48–72 hours as described (50). Secreted decorin was collected from confluent fibroblasts in 6-well plates; medium was changed to serum-free DMEM containing all other supplements 48 hours prior to collection (37). For FDC transdifferentiation, matched p5 cell populations were seeded on bovine aggrecan–coated 24-well plates (MilliporeSigma) at 400,000 cells per well in FDC differentiation medium: DMEM/F12 (Invitrogen), 10% FBS, antibiotic/antimycotic (Invitrogen), NEAA, 50 μg/ml L-ascorbic acid-2-phosphate (MilliporeSigma), 1% insulin/transferrin/selenite (Invitrogen), and 10 ng/ml TGFβ3 (R&D Systems) (24). Differentiation medium was changed every 2 days, and samples for protein and RNA analyses were obtained at days 1, 3, 5, and 7 and compared with samples obtained prior to plating (day 0). HEK293T cells were transfected with expression plasmids using polyethylenimine MW25000 (Polysciences). Cell viability/proliferation was determined using the WST-1 reagent as described (Roche Diagnostics) (51). Phenotypic rescue experiments were conducted by transfection of case A2 fibroblasts with expression vectors containing C-terminally myc-tagged GMAP-210 variants using FuGene HD according to the manufacturer’s instructions (Promega) or by electroporation, as described elsewhere (20).

### Plasmids, reagents, and antibodies.

The ORF of full-length *TRIP11* (Ensembl: ENST00000267622.8, GenBank: NM_004239.3) was cloned into the pcDNA5/FRT/TO vector (Thermo Fisher Scientific) carrying a carboxy-terminal myc-tag (20, 26). To obtain the *TRIP11*-ΔEx4 and the *TRIP11*-ΔEx9short variants, the 5′-end of full-length *TRIP11*-pCDNA5/FRT/TO was replaced with fragments generated by amplifying the respective region from patient cDNA by PCR and directional insertion of the DNA fragments via *Kpn*I and *Pac*I sites; an internal *Kpn*I restriction site in the WT *TRIP11* construct was eliminated by site-directed mutagenesis before directional cloning. Primer sequences are listed in Supplemental Table 3. To obtain the *TRIP11*-ΔEx9 variant containing a complete 5′-end, a digested DNA amplicon of exons 1–5 from WT cDNA replaced the 5′-end of the *TRIP11*-ΔEx9short variant. All generated plasmids were verified by complete bidirectional sequence analysis using primers listed in Supplemental Table 4.

The following antibodies were used: rabbit anti-TRIP11 (HPA002570, MilliporeSigma), mouse anti-TRIP11 (E-2, Santa Cruz Biotechnology Inc.), mouse anti-GAPDH (G-9, Santa Cruz Biotechnology Inc.), rabbit anti-GM130 (anti-N73pep) (52), mouse anti-GM130 (clone 35, BD Biosciences), rabbit anti-IFT20 (13615-1-AP, Proteintech), sheep anti-TGN46 (provided by Sreenivasan Ponnambalam, University of Leeds, Leeds, United Kingdom), sheep anti-ZFPL1 (53), mouse anti–GalNAc-T2 (provided by Henrik Clausen, University of Copenhagen, Denmark), mouse anti-myc (9B11, Cell Signaling Technology), goat anti-Myc (ab9132, Abcam), rabbit anti-GORAB (generated in house), sheep anti–golgin-84 (54), rabbit anti-LAMP1 (D2D11, Cell Signaling Technology), mouse anti-LAMP2 (H4B4, Developmental Studies Hybridoma Bank, University of Iowa, Iowa City, Iowa, USA), anti-decorin (14667, Proteintech), mouse anti-acetylated tubulin (clone 6-11B-1, MilliporeSigma), and mouse anti–α-tubulin (T9026, MilliporeSigma). Anti-MMP13 (MAB13424 and AB8114, MilliporeSigma; dilution 1:1,000), anti-COL10A1 (LS-C346132, LifeSpan Biosciences, dilution 1:1,000); and Alexa 488-, 546-, 594-, and 647-conjugated secondary antibodies were from Molecular Probes (Thermo Fisher Scientific). Horseradish peroxidase–conjugated secondary antibodies were from MilliporeSigma.

### RNA extraction and RT-PCR.

Total RNA from fibroblasts and cell lines was extracted with Trizol (Invitrogen), followed by DNaseI treatment (Roche Diagnostics) and column purification (Qiagen); cDNA was synthesized from 1 μg total RNA with Superscript III reverse transcriptase (Invitrogen), random hexanucleotides (MilliporeSigma), and oligo dT_16_ primers as described (29). Quantitative PCR (qPCR) was performed on a CFX384Touch Real-Time PCR System (Bio-Rad). 2^–ΔΔCt^ relative quantification, PCR efficiency correction, and multiple reference gene normalization were calculated with qBase (29). Oligonucleotides were designed using the Primer 3 software (http://primer3.ut.ee/) and are listed in Supplemental Table 5.

### Immunoblotting.

Fibroblasts were lysed using HMNT lysis buffer (20 mM HEPES-KOH [pH 7.4], 5 mM MgCl_2_, 0.1 M NaCl, 0.5% [wt/vol] Triton X-100) supplemented with protease inhibitor cocktail (Calbiochem). Total proteins from conditioned supernatants were precipitated by trichloroacetic acid (TCA) as described (37). Cell lysates (30 μg) were separated by electrophoresis in SDS-PAGE. Gels were then transferred onto a PVDF membrane (Amersham Hybond P 0.45, GE Healthcare) using a wet transfer system. The efficiency of transfer was assessed by Ponceau S stain (MilliporeSigma). Membranes were blocked by incubation in PBST buffer (PBS with 0.1% Tween-20) supplemented with 5% skimmed milk for 1 hour at room temperature (RT) and incubated with primary antibody solution (5% skimmed milk in PBST) overnight at 4°C. Membranes were washed 3 times in PBST buffer for 5 minutes each at RT (subsequent steps at RT) and incubated with secondary antibody solution (5% skimmed milk in PBST) for 1 hour. Membranes were washed 3 times in PBST buffer for 5 minutes and once in PBS for 5 minutes. The immunoreaction was detected with SuperSignal West Pico ECL chemiluminescent substrate (Thermo Fisher Scientific) using Bio-Rad Chemidoc MP Imager. Data were analyzed using Image Lab software (Bio-Rad). Analysis of molecular weight of GMAP-210 variants was conducted using a dedicated molecular weight analysis tool in Image Lab software (Bio-Rad) by comparing bands with known protein standards.

### Immunodetection of proteins and histochemistry.

Cells and supernatants were harvested 48–72 hours after transfection or at the indicated time points of the FDC protocol; prior to protein extraction, cells were washed twice with PBS. Lysates were prepared by incubation in 1 ml lysis buffer (20 mM Tris-HCl [pH 8], 137 mM NaCl, 10% glycerol, 1 mM NaF, 1 mM Na_3_VO_4_, 0.1% NP-40) plus complete protease inhibitor (Roche Diagnostics) for 20 minutes on ice, followed by centrifugation at 14,000 *g* and at 4°C for 10 minutes. For the analysis of secreted proteins, the medium was changed to serum-free Optimem I (Invitrogen), or serum-free DMEM containing all other supplements, 12–24 hours prior to collection. Pro-MMP13 (Quantikine DM1300, R&D Systems), COL10A1 (MBS917063, MyBiosource), pro-COL1A1 (ab210966, Abcam), and active caspase-3 (Quantikine KM130, R&D Systems) were quantified in conditioned supernatants and cell extracts by chromogenic ELISA as described (55).

Total GAG/proteoglycan content in FDC cultures was measured by an Alcian blue extraction method (40). Cells were carefully washed twice with PBS and fixed in 100% methanol for 30 minutes at –20°C, rinsed with distilled water, and incubated overnight at RT with staining solution (0.5% Alcian blue 8 GS [Carl Roth] in 1 N HCl). After extensive washing with distilled water, the dye was extracted with 500 μl of 6 M guanidine HCl in distilled water for 6 hours at RT. The optical density of the extract was determined by spectrophotometry at 630 nm (Dynatech). GAG values were normalized to cell numbers using WST-1, as described (51).

### Fluorescence microscopy.

Fibroblasts were seeded on glass coverslips and grown till 90% confluency. Cells were washed twice with PBS and fixed with 3% (wt/vol) paraformaldehyde (PFA) in PBS for 25 minutes at RT. Cells were then washed with PBS, and excess PFA was quenched with glycine. Cells were permeabilized by 4-minute incubation in 0.1% (wt/vol) Triton X-100 in PBS. Cells were incubated with primary antibody diluted in PBS for 1 hour at RT, washed 3 times with PBS, and then incubated for 1 hour with secondary antibody diluted in PBS, sometimes together with 100 ng/ml Hoechst 33342 dye (Thermo Fisher Scientific) to stain DNA. Following another 3 washes with PBS and 2 in ddH_2_0, coverslips were mounted in Mowiol 4-88. Images were acquired using an Olympus BX60 upright microscope equipped with a MicroMax cooled, slow-scan CCD camera (Princeton Instruments) driven by Metaview software (University Imaging Corporation). Images were processed using ImageJ software (NIH).

### Electron microscopy.

Cells were grown on 10-cm dishes until they reached 80% confluency. The samples were fixed with 4% formaldehyde + 2.5% glutaraldehyde in 0.1 M HEPES buffer (pH 7.2), and cells were scraped and centrifuged at 5,200 *g* for 10 minutes. Cells were post-fixed with 1% osmium tetroxide + 1.5% potassium ferrocyanide in 0.1 M cacodylate buffer (pH 7.2) for 1 hour and in 1% uranyl acetate in water for 1 hour. The samples were dehydrated in ethanol series infiltrated with low-viscosity resin (TAAB) and polymerized for 24 hours at 60°C. Sections of 70 nm were cut with an Ultracut ultramicrotome (Reichert) and observed with a FEI Tecnai 12 Biotwin microscope at 100 kV accelerating voltage. Images were taken with a Gatan Orius SC1000 CCD camera. Quantification of cells with large vesicular profiles, defined as peri-Golgi circular profiles with a diameter of >100 nm, were conducted manually in a blinded manner.

### Mitochondrial relocation assay.

The mitochondrial relocation assay was performed as previously described (20). Briefly, HeLaM cells were cotransfected with plasmids carrying TRIP11-mycFKBP variants and mito-FRB plasmid (a gift from Stephen Royle, University of Warwick, Warwick, United Kingdom) using FuGene HD (Promega). The following day, HeLaM cells were treated with 2.5 μg/ml nocodazole (MilliporeSigma) for 2 hours, followed by addition of 1 μM rapamycin (Calbiochem) for 3 hours to induce targeting of TRIP11-mycFKBP onto mitochondrial outer membranes. Cells were fixed and processed for immunocytochemistry as described above.

### Pulse-chase analysis of protein secretion.

Analysis of protein secretion was performed using a pulse-chase approach as described previously (34). Cells were seeded onto 3.5-cm dishes and grown until they reached 95% confluency. Cells were starved for 1 hour in methionine- and cysteine-free DMEM (Invitrogen) supplemented with 10% (vol/vol) FBS and 1 mM L-glutamine and pulsed for 20 minutes with fresh starvation medium containing 25 mCi/ml [^35^S]Met and [^35^S]Cys protein labeling mix (PerkinElmer). After washing with PBS, cells were incubated in chase medium (DMEM, 10 mM HEPES [pH 7.4], 1 mM methionine, 1 mM cysteine, 100 μg/ml bovine serum albumin) for 0, 30, or 60 min. Proteins secreted to the medium were precipitated using TCA, while cells were lysed using HMNT lysis buffer. Cells and medium fractions were subjected to SDS-PAGE; gels were fixed for 10 minutes in 20% (vol/vol) methanol and 10% (vol/vol) acetic acid and dried for 2 hours at 65°C using a gel dryer (Bio-Rad). Dried gels were exposed to phosphor-imaging plates and developed after 10 days using a fluorescent image analyzer (FLA 7000, Fujifilm). Signals from radiolabeled proteins were quantified using AIDA software (Elysia-raytest).

### Statistics.

Statistical analyses were conducted with use of GraphPad Prism software (GraphPad Software). D’Agostino-Pearson and Shapiro-Wilk tests were used for comparison of the distribution of data with a Gaussian distribution. Depending on the result, a Mann-Whitney *U* test or 1- or 2-way ANOVA with Bonferroni’s correction for multiple comparisons was performed. The number of cells with large vesicular profiles and the Golgi morphology classes in the rescue experiments of Golgi compaction/fragmentation in case A2 fibroblasts were compared using a χ^2^ test. Amounts of secreted proteins were compared using 1-way ANOVA followed by Dunnett’s multiple comparisons test. Statistical significance cut-offs were set as follows: *P ≤ 0.05, **P < 0.01, ***P < 0.001, and ****P < 0.0001.

### Study approval.

All individuals in our study were recruited by physician-initiated referral. The study was conducted in accordance with the Declaration of Helsinki protocols and approved by the institutional ethics review board of Freiburg University Hospital, Germany (Ethik-Kommission der Albert-Ludwigs-Universität Freiburg Nr. 349/13). Written informed consent for skin and tissue biopsies and molecular studies was obtained from the affected individuals and/or their legal guardians in accordance with current German law (GenDG). Control samples and primary cells were collected from ancestry-, sex-, and age-matched healthy individuals under the same criteria and regulations.

## Author contributions

AW, TMW, J. Schneider, JH, TW, MS, GJP, and EL conducted experiments and acquired data. JAF, MS, and GJP contributed data and reagents. SU, VF, MH, NV, KS, BZ, J. Spranger, ASF, EL, ABZ, and GN contributed clinical data. J. Spranger, GN, SU, ASF, and EL analyzed clinical data. AW, TMW, ML, and EL designed research studies and analyzed data. AW, TMW, ML, and EL wrote and edited the manuscript; SU and J. Spranger edited the manuscript. All coauthors read and approved of the manuscript.

## Supplementary Material

Supplemental data

## Figures and Tables

**Table 3:**
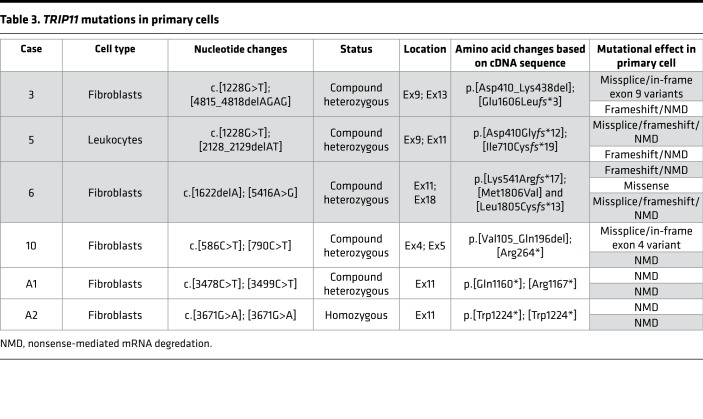
*TRIP11* mutations in primary cells

**Table 2:**
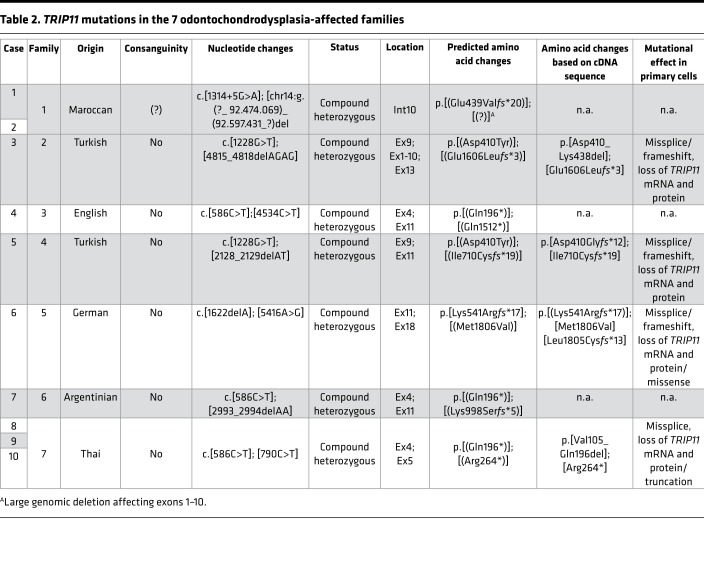
*TRIP11* mutations in the 7 odontochondrodysplasia-affected families

**Table 1:**
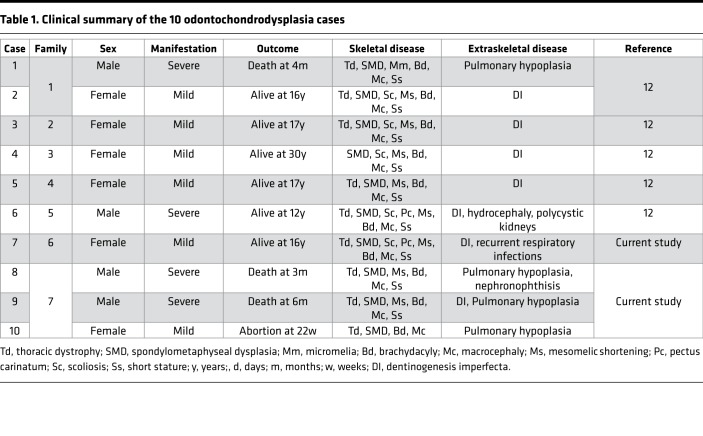
Clinical summary of the 10 odontochondrodysplasia cases

## Version 1 02/07/2019

Electronic publication
