# Supplementary material for: Hypomorphic mutations of TRIP11 cause odontochondrodysplasia
Source: JCI Insight. 2019 Feb 7;4(3):e124701. doi: 10.1172/jci.insight.124701 (PMC6413787; doi:10.1172/jci.insight.124701)
Supplement: Supplemental data [file jciinsight-4-124701-s024.pdf]

Supplemental Information

Hypomorphic mutations of *TRIP11* cause Odontochondrodysplasia

Anika Wehrle and Tomasz M. Witkos *et al.*

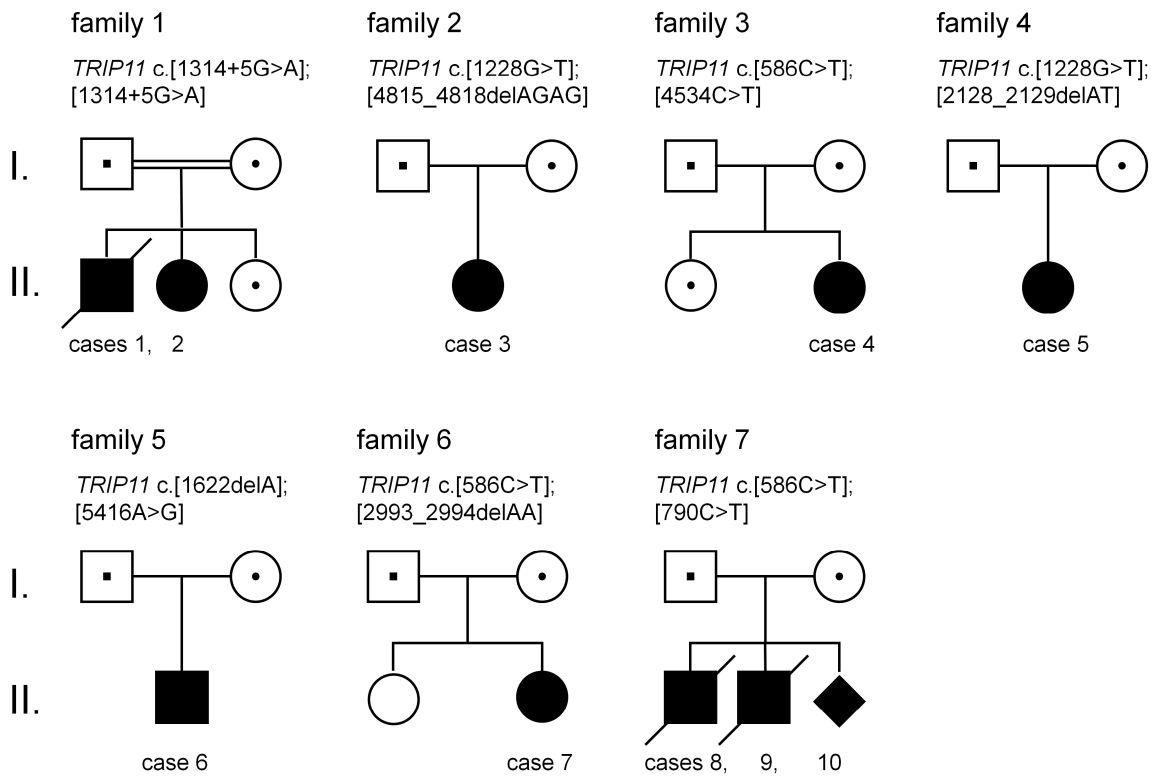

Supplemental Figure 1: Abridged pedigrees of ODCD families.

Supplemental Table 1: *TRIP11* mutations in the five achondrogenesis 1A (ACG1A) families.

Family	Origin	Consan-guinity	Affected Children	Nucleotide Changes	Status	Location	Predicted Amino Acid Changes	Amino Acid changes based on cDNA sequence	Mutational effect in primary cells
A1	Italian	no	1	c.[3478C>T]; [3499C>T]	compound heterozygous	Ex11	p.[(Gln1160*)]; [(Arg1167*)]	p.[Gln1160*]; [Arg1167*]	truncation, loss of *TRIP11* mRNA and protein
A2	Italian	yes	1	c.[3671G>A]; [3671G>A]	homozygous	Ex11	p.[(Trp1224*)]; [(Trp1224*)]	p.[Trp1224*]; [Trp1224*]	truncation, loss of *TRIP11* mRNA and protein
A3	Finnish	no	1	c.[3962T>A]; [3962T>A]	homozygous	Ex11	p.[(Leu1321*)]; [(Leu1321*)]	p.[Leu1321*]; [Leu1321*]	truncation, loss of *TRIP11* mRNA and protein
A4	American	no	1	c.[790C>T]; [4127C>A]	compound heterozygous	Ex5; Ex11	p.[(Arg264*)]; [(Ser1376*)]	n.a.	n.a.
A5	German	no	2	c.[2467_2470delAGAA]; [5162_5260del]	compound heterozygous	Ex11; Ex16	p.[(Arg823Val*fs**15)]; [(Glu1721_Gln1753 delinsAsn1754Met*fs**15)]	n.a.	n.a.

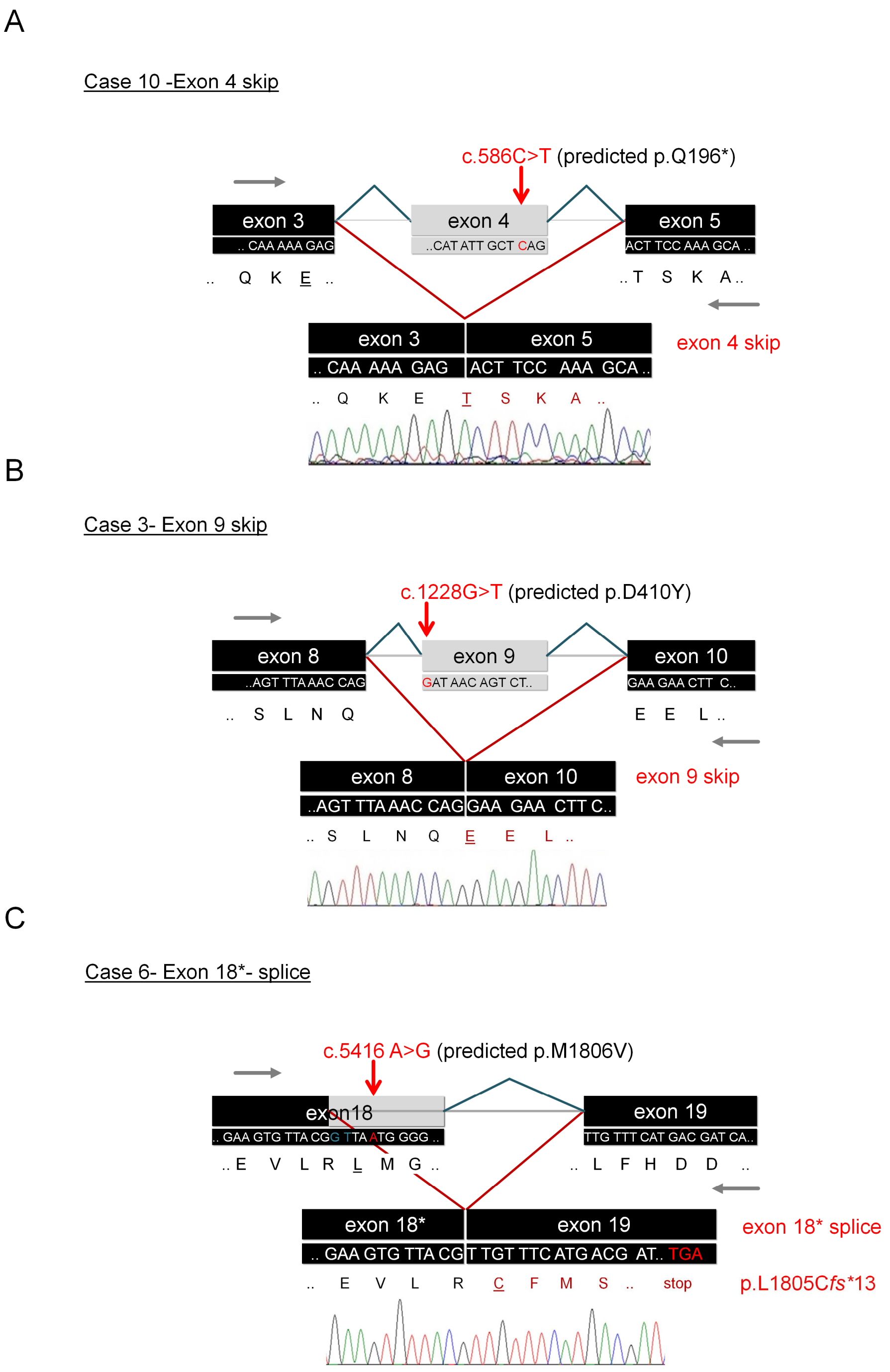

Supplemental Figure 2: Aberrant splicing of *TRIP11* in odontochondrodysplasia (ODCD). Exon-spanning reverse transcription PCR analysis of *TRIP11* using cDNA derived from patient and control (CTL) fibroblasts; schematic representation of the amplified and sequenced regions. Grey horizontal arrows indicate the relative position of primers; mutations are indicated by red vertical arrows; exons are not drawn to scale; amino acids of the new reading frames are indicated in dark red; substitutions and the replaced wild-type amino acids are underlined. M: marker; bp: base pairs; **(A)** The c.586C>T *TRIP11* mutation causes aberrant splicing and in-frame skipping of exon 4. **(B)** In case 3, the recurrent c.1228G>T (p.Asp410Tyr) predicted missense mutation in fact abrogates the splice acceptor site resulting in an in-frame deletion of exon 9. **(C)** The c.5416A>G, p.Met1806Val, predicted missense mutation in case 6 generates an ectopic exonic splice donor site of exon 18, causing a frameshift and premature stop (p.Leu1805Cys*fs**13).

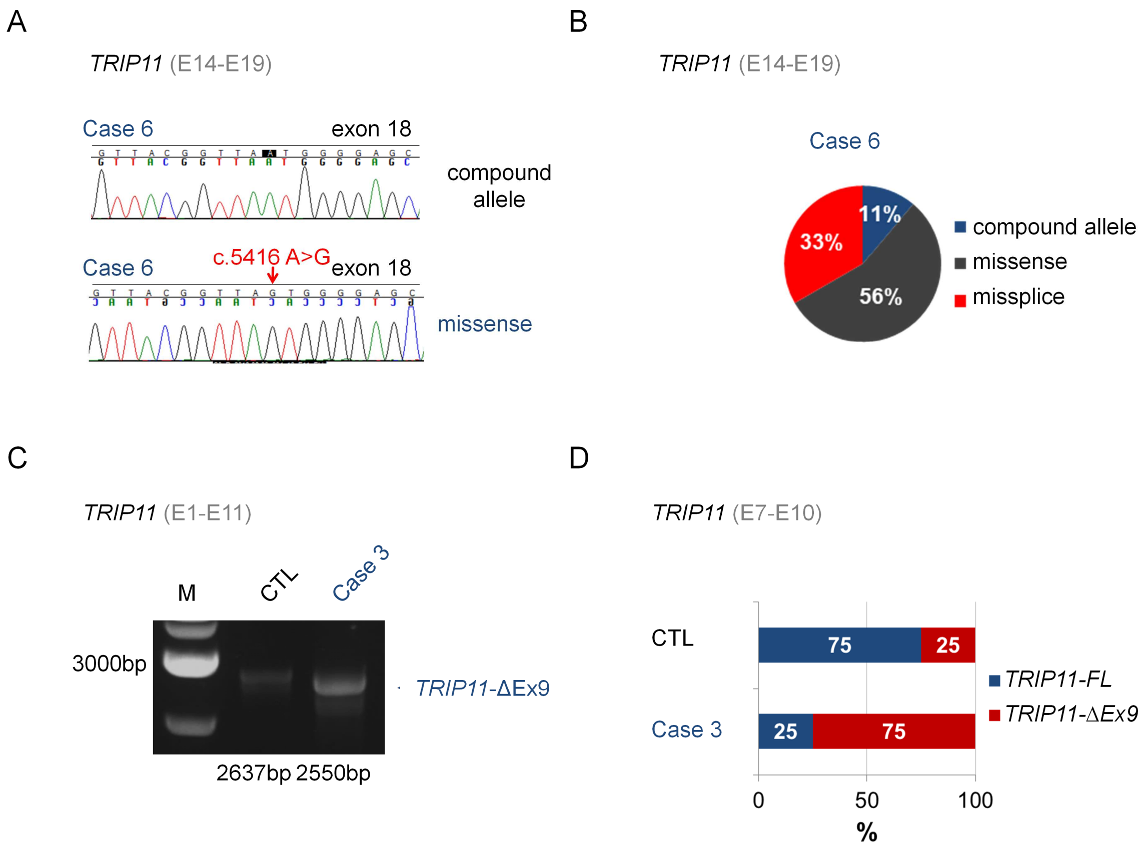

Supplemental Figure 3: Variable splicing of *TRIP11* in odontochondrodysplasia (ODCD). (A) Electropherograms of subcloned and sequenced *TRIP11* cDNA amplicons derived from primary fibroblasts of case 6. Sequence analysis reveals also transcripts with the c.5416A>G transition. **(B)** Variable splicing of exon 18. The diagram shows the fraction of subcloned and sequenced *TRIP11* cDNA amplicons (n=10) derived from primary fibroblasts of case 6 (missense, missplice, and amplicons of the compound allele). **(C)** Structure of the ODCD-associated *TRIP11*-ΔEx9 mRNA isoform. A reduced PCR product size (2550bp compared to 2637bp) indicates skipping of exon 9, but a normal 5'-end, and was detected in case 3 by reverse transcription PCR analysis of *TRIP11* cDNA derived from fibroblasts. Primers comprising regions upstream of exon 9 (spanning exons 1 to 11 of *TRIP11*) were used (E1-E11, relative primer positions are indicated as grey arrows in Figure 2). **(D)** Variable splicing of the *TRIP11*-ΔEx9 isoform in patient 3 and control fibroblasts. The bar graph indicates the fraction of subcloned and sequenced *TRIP11* cDNA amplicons. Clones with (*TRIP11*-FL), or without exon 9 (*TRIP11*-ΔEx9) were detected using primers spanning exon 7 to 10 of *TRIP11* (E7-E10), relative primer positions are indicated as grey arrows in Figure 2D. Results are based on the sequence analysis of 26 (CTL) and 13 (case 3) individual clones.

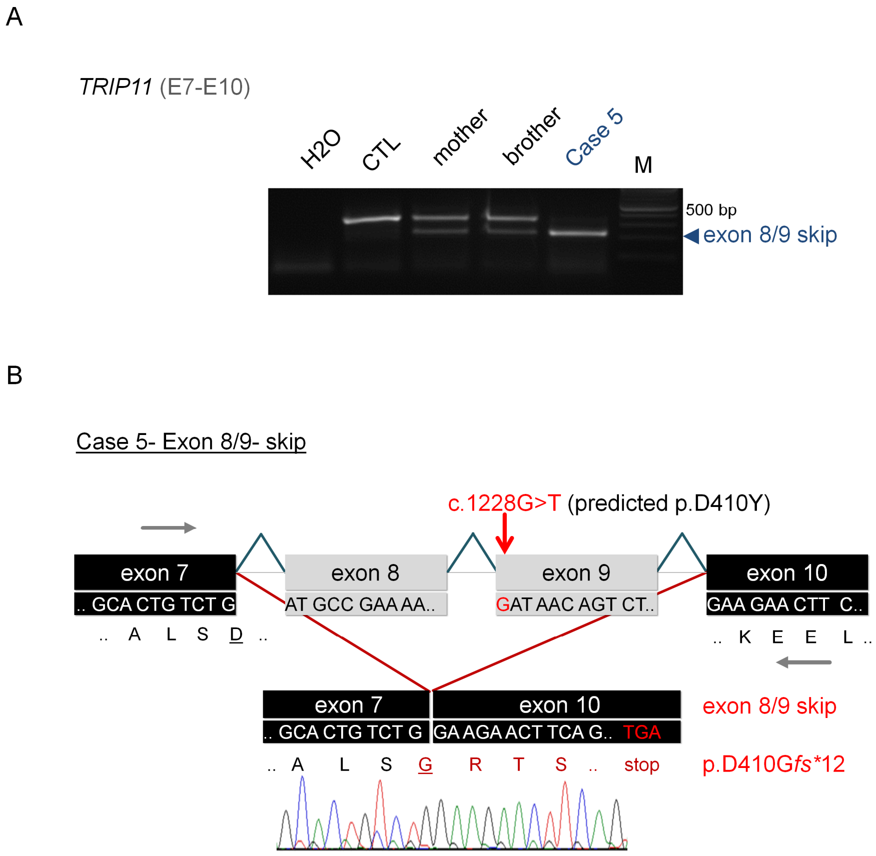

Supplemental Figure 4: Cell type-dependent splicing of *TRIP11* in odontochondrodysplasia (ODCD). (A) Cell type-dependent missplicing caused by the c.1228G>T (*TRIP11*-ΔEx9) mutation. Exon-spanning reverse transcription PCR analysis of *TRIP11* using cDNA derived from white blood cells of case 5. The resulting missplice joins exon 7 with exon 10, as shown by the smaller cDNA amplicon in the patient and in her heterozygous relatives with primers flanking the skipped exons. Of note, the larger band representing the wild-type allele is absent in case 5, indicating that the compound heterozygous paternal mutation c.2128_2129delAT (p.Ile710Cys*fs**19) induces mRNA degradation. **(B)** Sanger sequencing of the cDNA amplicon confirms skipping of exons 8 and 9, resulting in a frameshift and premature stop (p.Asp410Gly*fs**12, bottom panel). The mutation site is indicated by a red vertical arrow; grey horizontal arrows indicate the relative position of primers; exons are not drawn to scale; amino acids of the new reading frame are indicated in red; substitutions and the replaced wild-type amino acids are underlined.

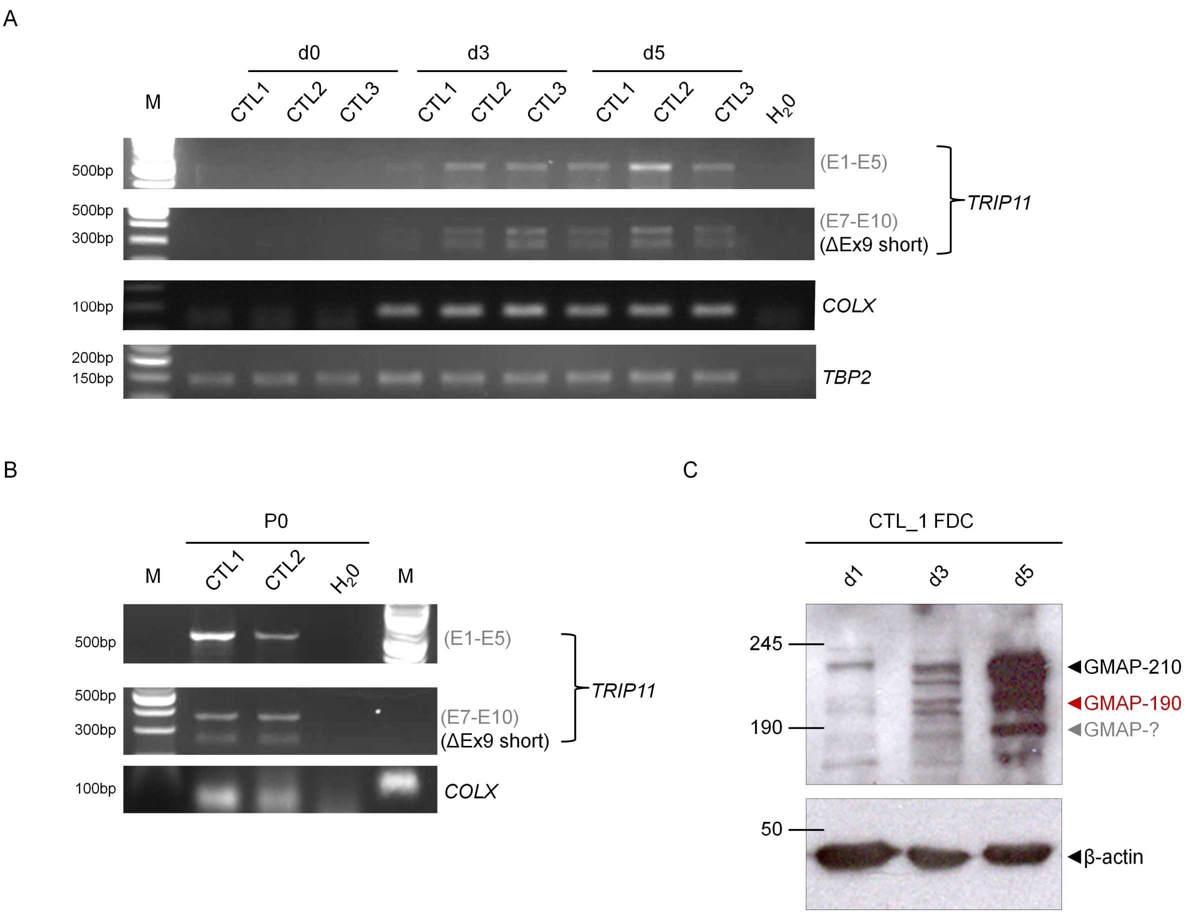

Supplemental Figure 5: Expression of *TRIP11* mRNA isoforms in wild-type chondrogenic cells and primary chondrocytes. (A) Semi-quantitative reverse transcription (RT)-PCR analysis of *TRIP11* and *COL10A1* using cDNA of three independent wild-type fibroblast-derived chondrocyte (FDC) cultures (CTL1 to 3) at days 0 to 5 (d0-d5) of chondrogenic differentiation; *TBP2* was used as a reference housekeeping mRNA. **(B)** RT-PCR analysis of *TRIP11* and *COL10A1* using cDNA isolated from human primary articular chondrocytes of two healthy donors. Primers E1-E5 detect full-length *TRIP11*-FL at 505bp, primers E7-E10 the isoform *TRIP11*-ΔEx9short at 275bp (see also the schematic of *TRIP11* isoforms in Figure 2D). **(C)** Western blot analysis of GMAP-210 isoforms in human FDC cells using an antibody directed against the carboxy-terminus of the protein. In addition to GMAP-210, which migrates at approximately 230kDa, a double band is observed at approximately 210kDa and may thus represent GMAP-190. Another, yet uncharacterized, GMAP-? protein with an apparent molecular weight of 185 to 190kDa is induced in late-stage FDC cells; β-actin was used as a loading control.

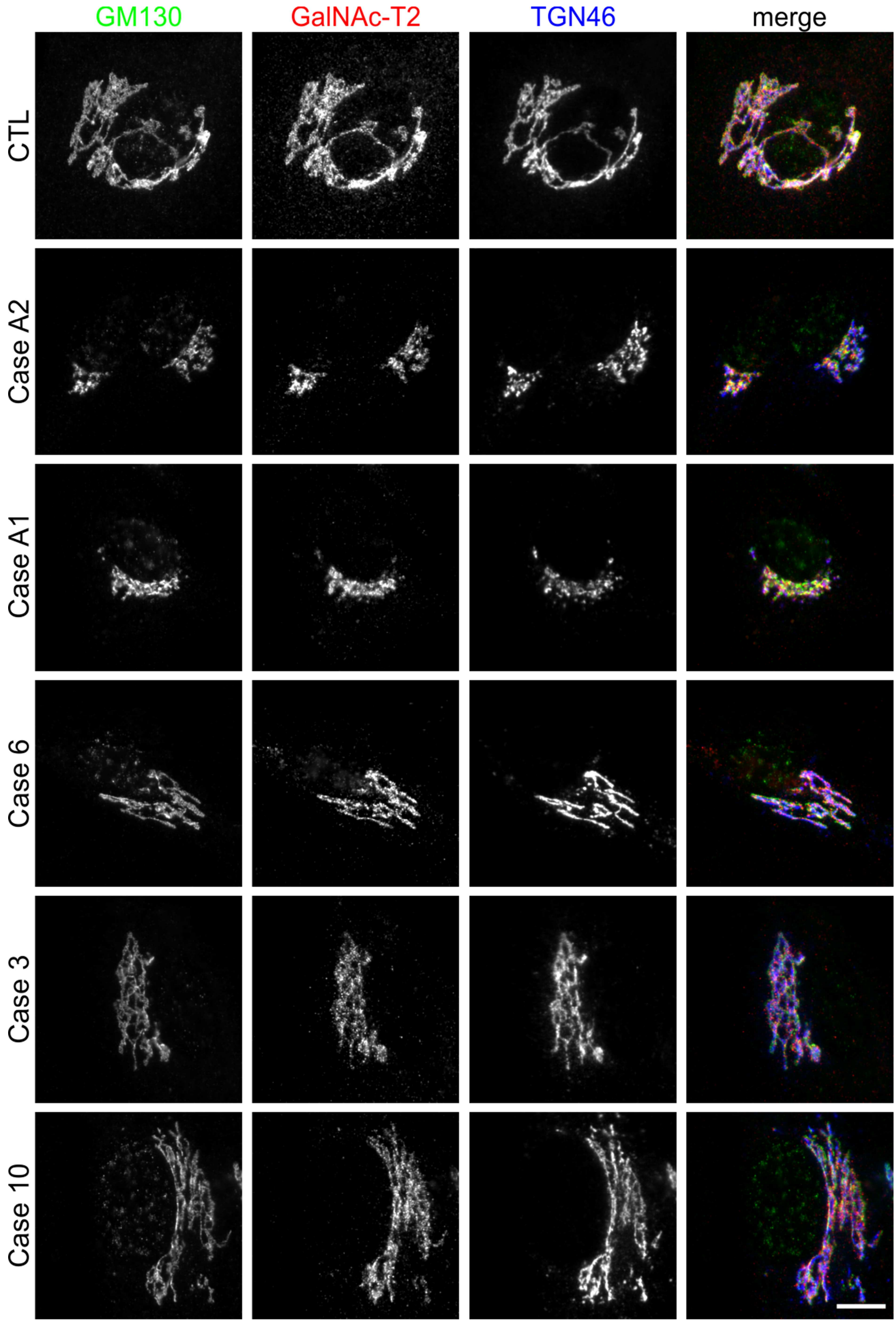

Supplemental Figure 6: Golgi ribbon morphology is preserved in odontochondrodysplasia (ODCD). Wide-field microscopy of control (CTL) and patient-derived fibroblasts co-stained with antibodies directed against the *cis*-Golgi protein GM130 and antibodies directed against the Golgi-resident enzyme GalNAc-T2 and the *trans*-Golgi network protein TGN46. Scale bar: 10µm.

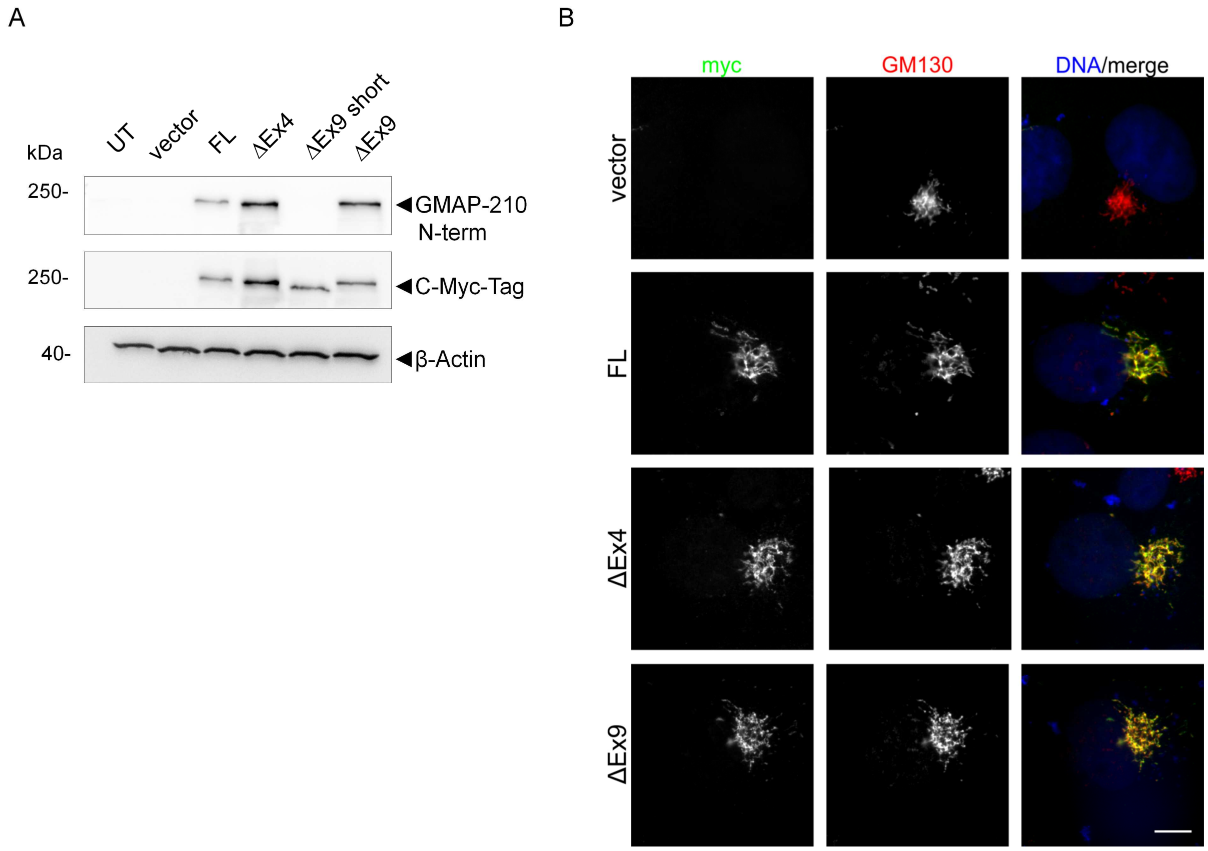

Supplemental Figure 7: Golgi targeting of GMAP variants. (A) Western blot analysis of whole-cell lysates of untransfected HEK-T control cells or transiently transfected with vectors expressing different variants of carboxy-terminally myc-tagged GMAP proteins. Beta-actin was used as a loading control. Endogenous GMAP, which is less abundant than transfected protein, is not detected with anti-GMAP-210 antibodies due to low exposure time. UT: untransfected parental cells; vector: empty vector; FL: myc-tagged full-length GMAP-210; ΔEx4: myc-tagged GMAP lacking exon 4; ΔEx9 short: myc-tagged GMAP with shorter amino-terminus lacking exon 9; ΔEx9: myc-tagged GMAP with complete amino-terminus lacking exon 9. **(B)** Immunofluorescence analysis of COS-7 cells transiently transfected with above vectors expressing carboxy-terminally myc-tagged GMAP variants. Golgi targeting was assessed by staining with anti-myc and analyzing co-localization with the *cis*-Golgi marker GM130. Scale bar: 10µm.

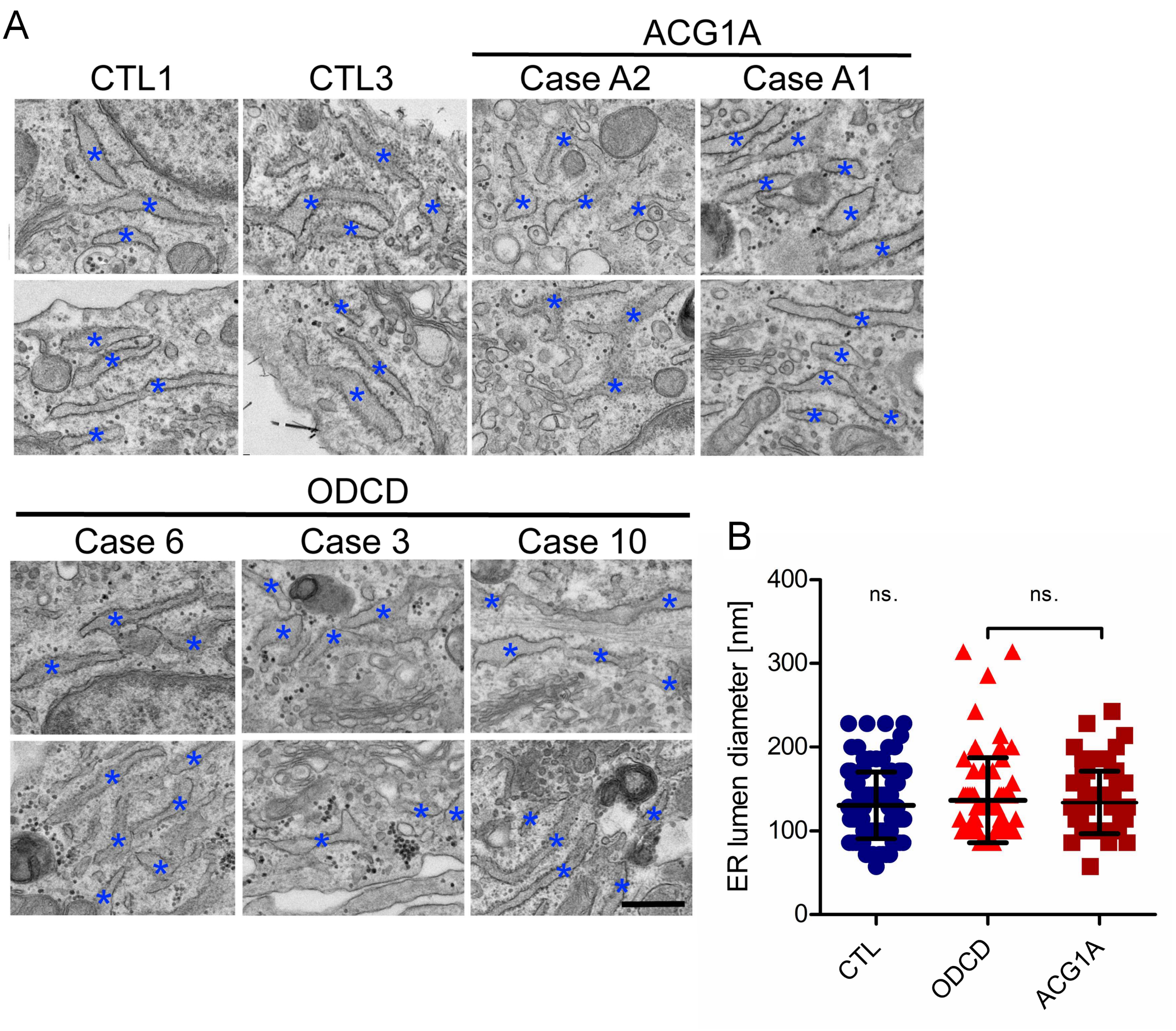

Supplemental Figure 8: Transmission electron microscopy of the endoplasmic reticulum (ER) in achondrogenesis 1A (ACG1A) and odontochondrodysplasia (ODCD). (A) Representative sections through the ER at the indicated sites (blue asterisks) of control (CTL_1 and CTL_3), ACG1A (cases A2 and A1) and ODCD (cases 6, 3 and 10) fibroblasts; scale bar: 500nm. **(B)** The diameter of the ER lumen was measured in 8 to10 cells per case (>50 measurements). Data means are indicated as horizontal lines, error bars represent ± SD; differences were assessed by one-way ANOVA with Dunn's multiple comparisons test; ns., not statistically significant.

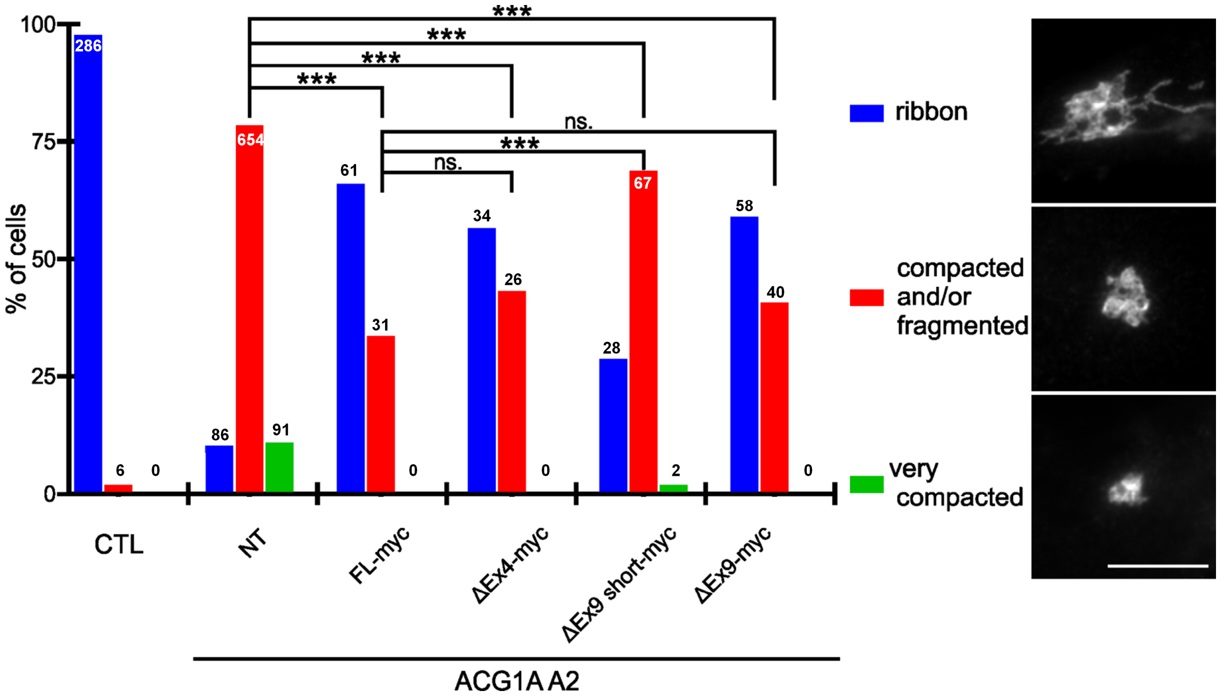

Supplemental Figure 9: Rescue of Golgi morphology by GMAP variants. Wide-field microscopy of GMAP-210-deficient fibroblasts (case A2) transfected with the indicated myc-tagged GMAP expression constructs. Golgi morphology was assessed by GM130 co-immunostaining; relative number of cells exhibiting restored Golgi ribbon structure (indicated in blue), compacted and/or fragmented Golgi apparatus (indicated in red) or very compacted Golgi apparatus (indicated in green); CTL: control wild-type fibroblasts, NT: not transfected parental ACG1A cells, FL-myc: full length myc-tagged GMAP-210, ΔEx4: GMAP-200 (lacking exon 4), ΔEx9: GMAP-207 (complete amino-terminus and lacking exon 9), ΔEx9short: GMAP-190 (short amino-terminus and lacking exon 9). Scale bar: 10µm. Bar graphs represent quantifications from three independent experiments (n>100, chi-square test; ns. not statistically significant, *** p<0.001).

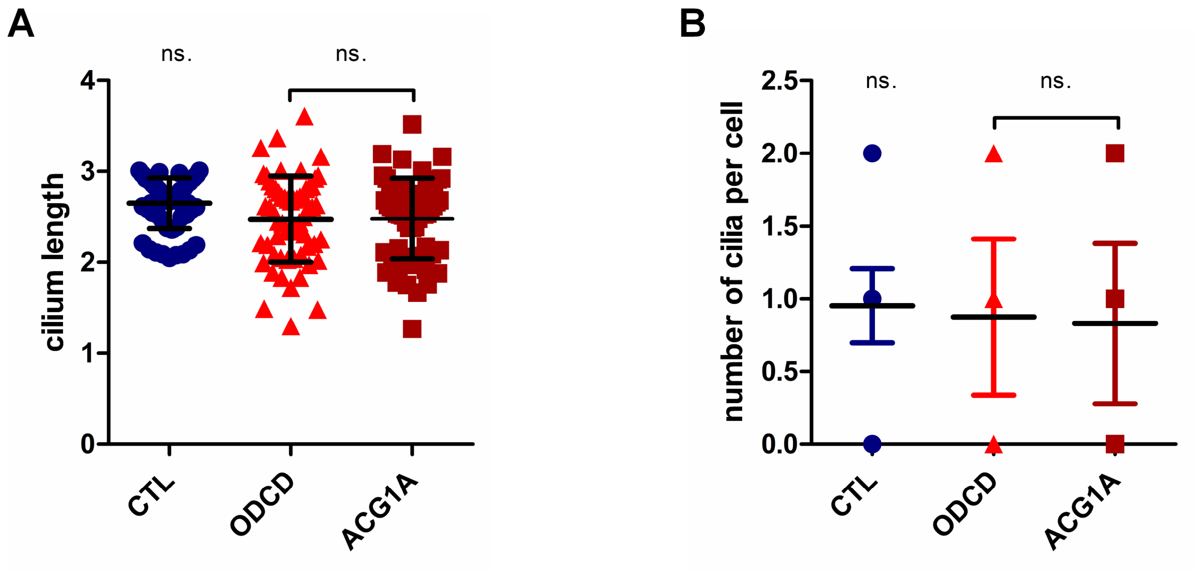

Supplemental Figure 10: Ciliary phenotypes in achondrogenesis 1A (ACG1A) and odontochondrodysplasia (ODCD). (A) Mean cilia length and **(B)** number of cilia per cell in serum-starved fibroblasts of patients and controls stained with anti-acetylated tubulin. Both longer and shorter cilia occur more often in ACG1A and ODCD patient-derived cells than in matched controls (n=105) as indicated by larger standard deviations of the mean cilum length, but differences do not reach statistical significance. Data means are indicated as horizontal lines, error bars represent ± SD; differences were assessed by one-way ANOVA with Dunn's multiple comparisons test; ns., not statistically significant.

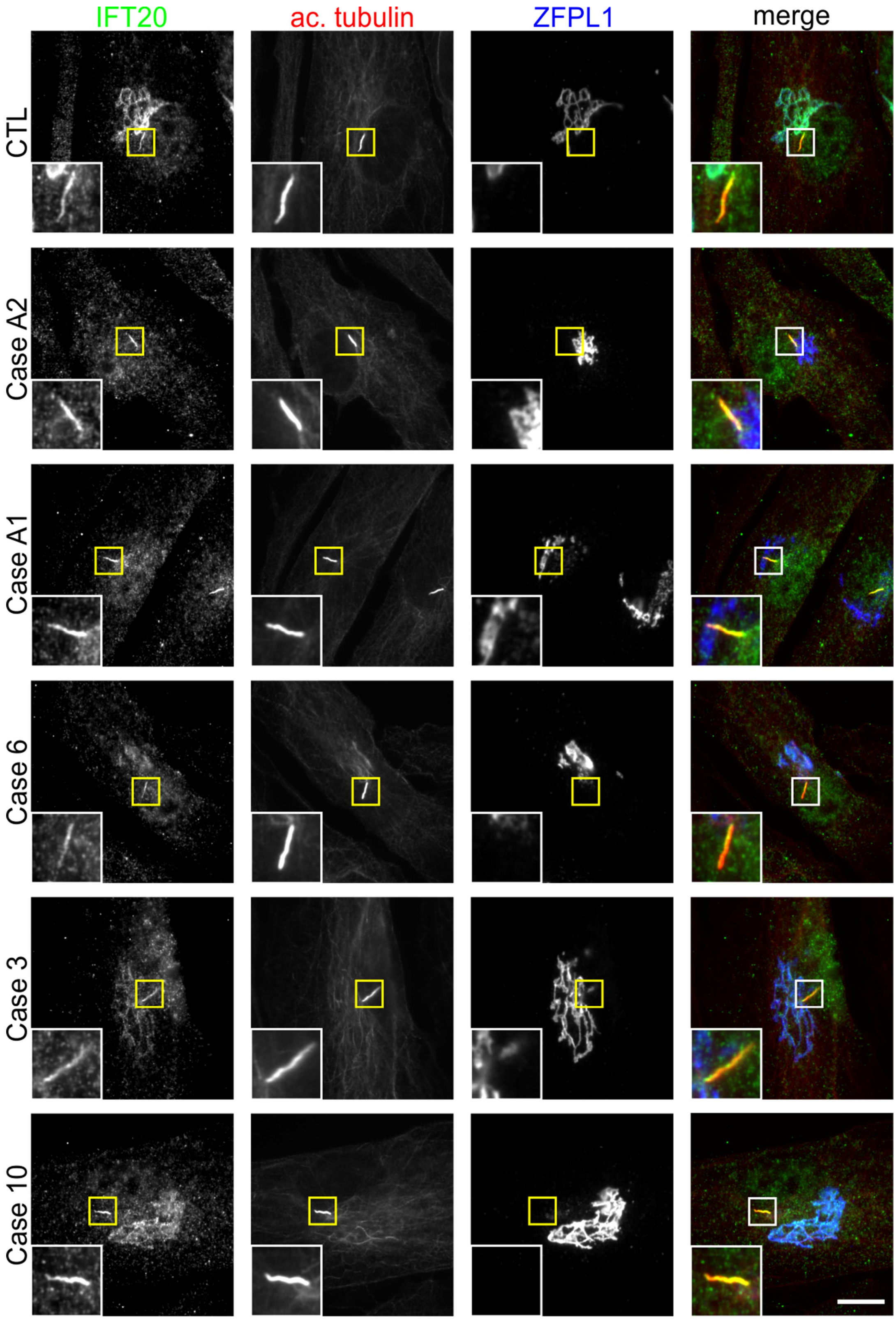

Supplemental Figure 11: Ciliary IFT20 in achondrogenesis 1A (ACG1A) and odontochondrodysplasia (ODCD). Wide-field microscopy of GMAP-210-deficient serum-starved primary cells co-stained with antibodies against acetylated tubulin, IFT20, and ZFPL1, a membrane protein at the *cis*-Golgi apparatus. Insets (bottom left corner) show zooms of corresponding areas marked by a yellow square. Scale bar: 10µm.

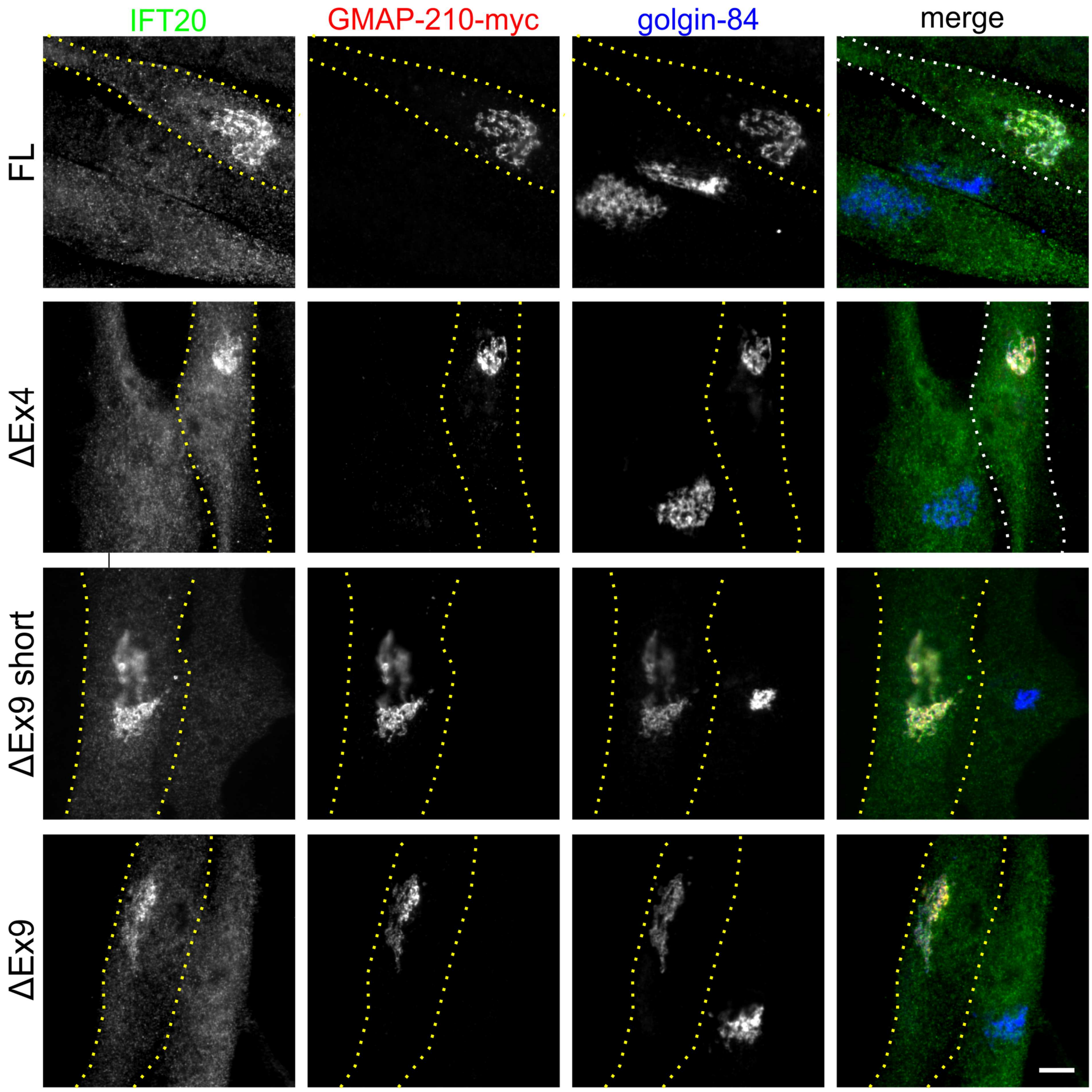

Supplemental Figure 12: Odontochondrodysplasia (ODCD)-associated GMAP variants target IFT20 to the Golgi. Transient overexpression of myc-tagged GMAP expression constructs in GMAP-210-deficient fibroblasts (case A2). The boundaries of transfected cells are outlined by yellow dotted lines, adjacent non-transfected cells serve as negative controls. Fibroblasts were stained with the indicated antibodies and analyzed by wide-field microscopy. Golgi targeting was assessed by co-localization with golgin-84. Scale bar: 10µm.

A

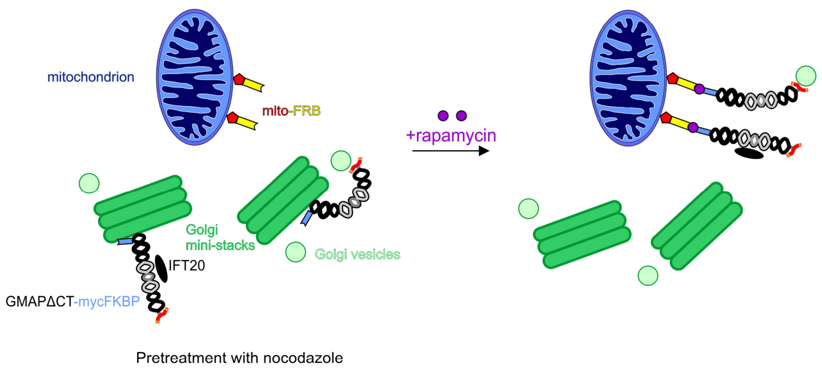

B

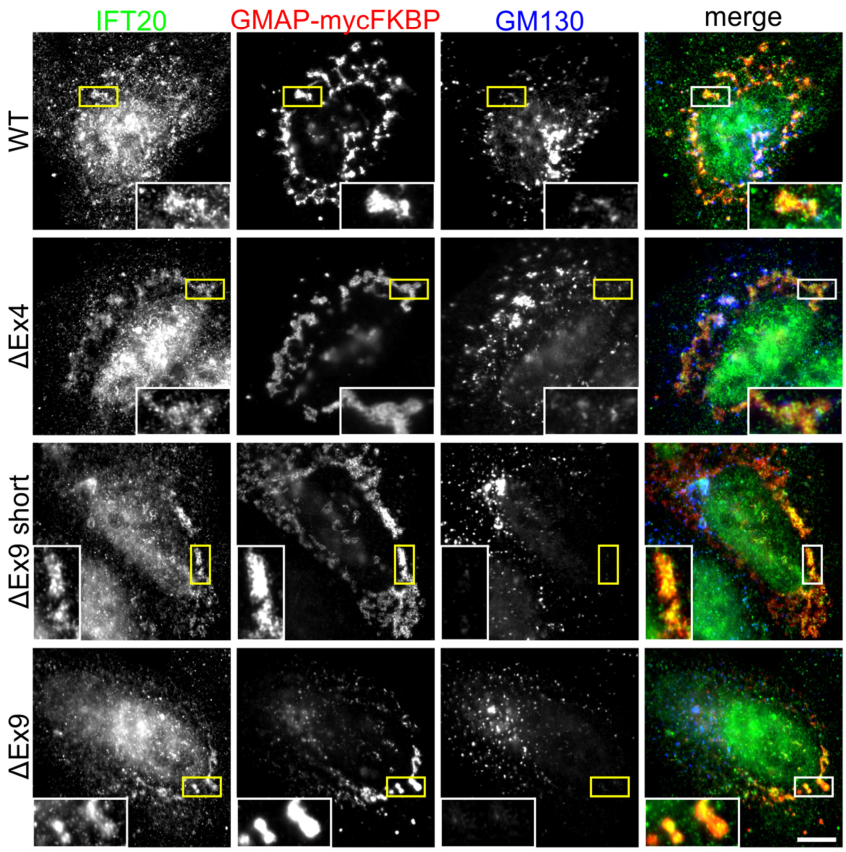

Supplemental Figure 13: Odontochondrodysplasia (ODCD)-associated GMAP variants are competent to bind IFT20 *in vivo*. (A) Schematic of the mitochondrial relocation assay. The carboxy-terminal region of GMAP-210 containing the GRIP-related Arf-binding (GRAB) domain is replaced and fused to FK506-binding protein (FKBP, GMAPΔCT-mycFKBP, indicated in light blue) and co-expressed in cells with mitochondrially localized translocator of outer membrane 70p (Tom70p) fused to FKBP-rapamycin binding domain (FRB, mito-FRB, indicated in yellow and red, respectively). GMAPΔCT-mycFKBP variants are rapidly relocated from the Golgi to mitochondria upon rapamycin-induced hetero-dimerization of the FKBP and FRB domains. HeLaM cells co-expressing GMAPΔCT-mycFKBP variants and mito-FRB were pretreated with nocodazole to disperse Golgi vesicles, induced with rapamycin, fixed, and **(B)** co-stained for IFT20 (green), the myc epitope (red) to detect the reroutable GMAP-210 and the *cis*-Golgi resident GM130 (blue). Scale bar: 10µm.

A

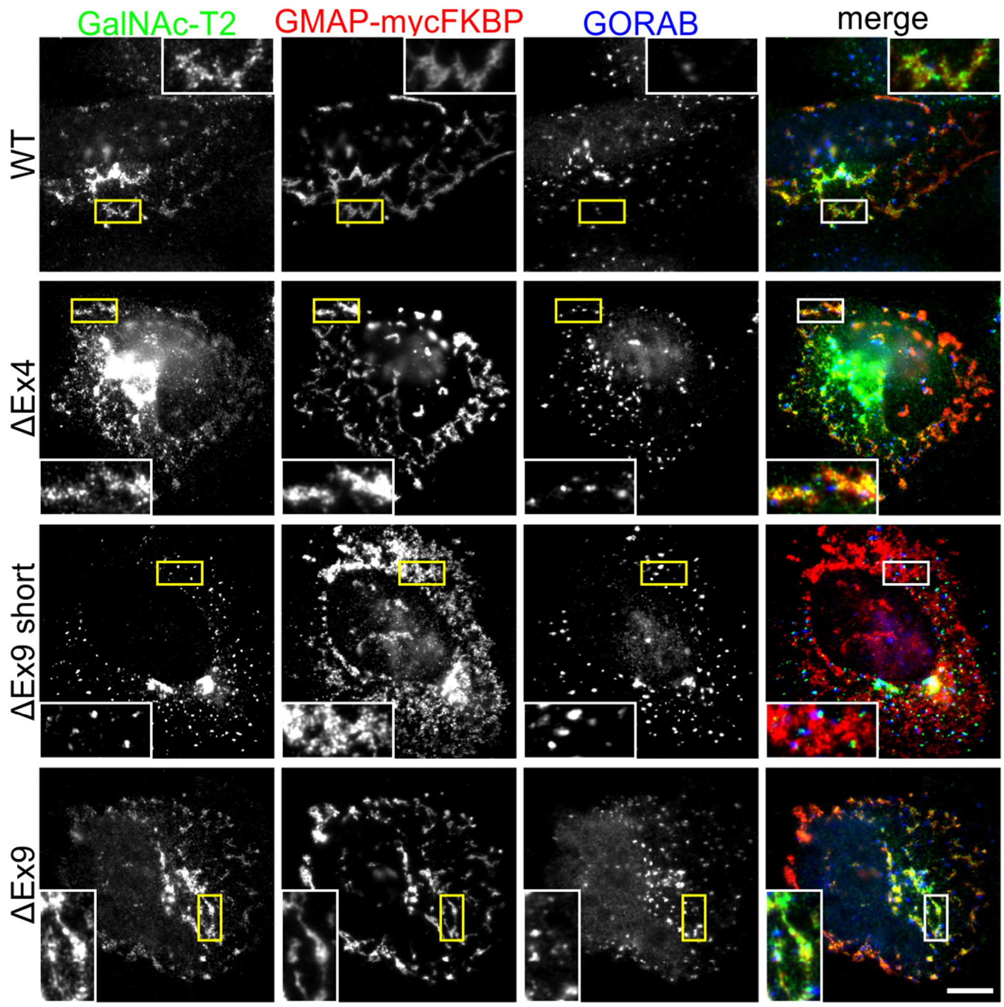

B

		Relocation of:	
GMAP variant	Graphical representation	IFT20	GalNAc-T2
WT	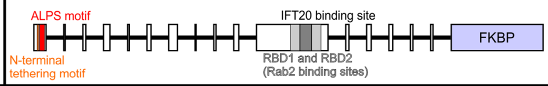	✔	✔
ΔEx4	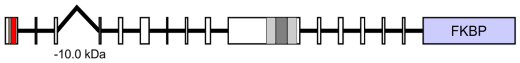	✔	✔
ΔEx9 short	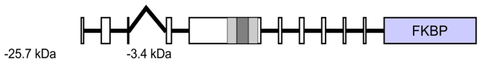	✔	✖
ΔEx9	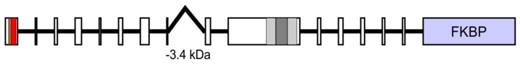	✔	✔

Supplemental Figure 14: Odontochondrodysplasia (ODCD)-associated GMAP variants are competent to tether transport vesicles *in vivo*. (A) HeLaM cells were co-stained for the Golgi-resident enzyme GalNAc-T2 (green), the myc epitope (red) to detect the reroutable GMAP variants, and the *trans*-Golgi protein GORAB (blue). Scale bar: 10µm. **(B)** Graphical representation of the analyzed GMAP variants and summary of results of the relocation assay.

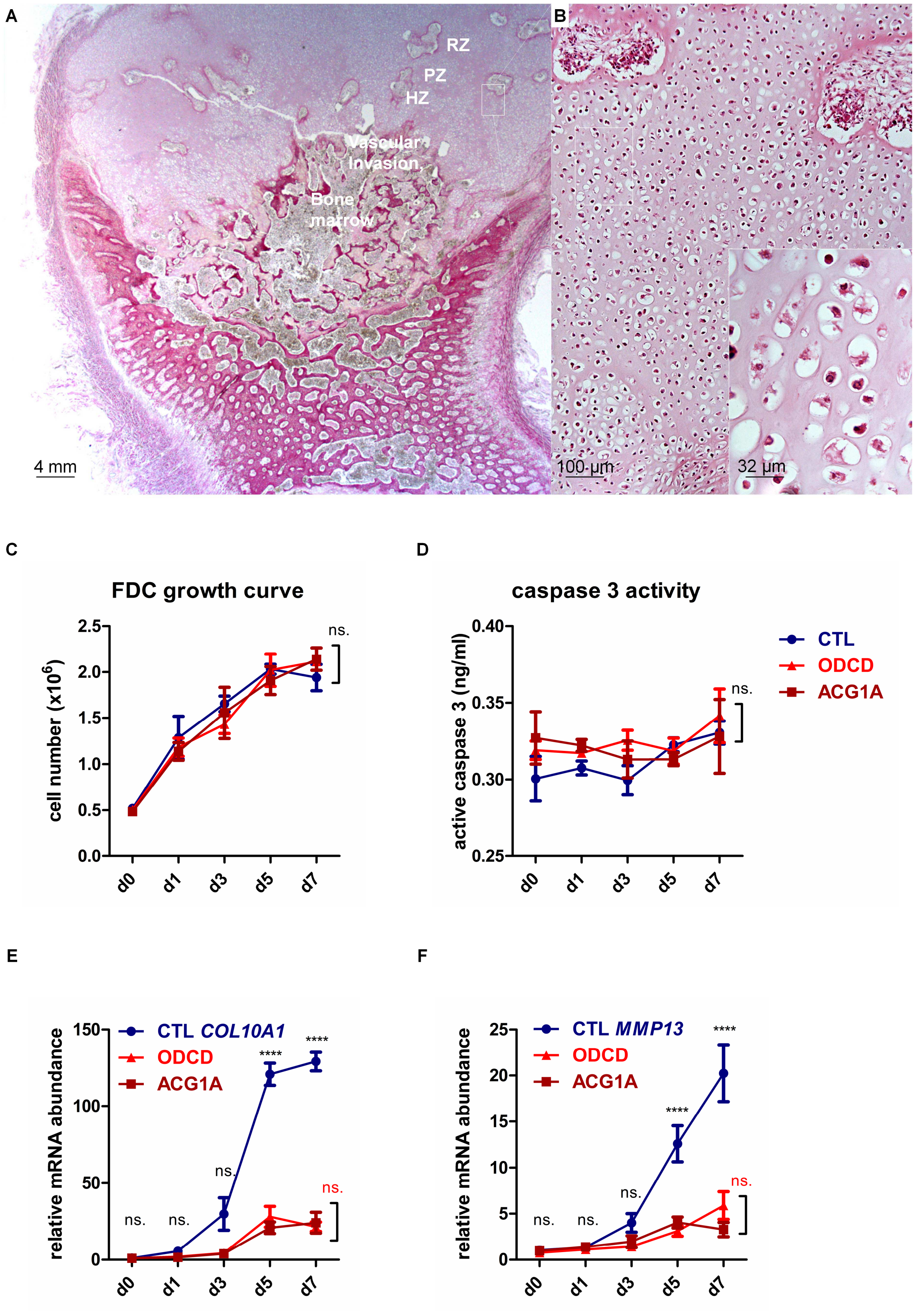

Supplemental Figure 15: Normal proliferation and apoptosis, but impaired hypertrophic differentiation in *TRIP11*-mutant human chondrogenic cells. (A) The human growth plate in ACG1A at gestational week 22. Hematoxilin/eosin-stained longitudinal section of the distal femur epiphysis of case A5, showing reduced cartilage extracellular matrix (ECM) and **(B)** hyper-cellularity in the reserve (RZ) and proliferating zones (PZ). No columnar alignment of

chondrocytes is seen in the pre-hypertrophic and hypertrophic zones (HZ) which are not clearly definable and characterized by the accumulation of disordered larger chondrocytes with cytoplasmic inclusions, as seen by larger magnification in the inset of (B); scale bars indicate 4mm, 100µm, and 32µm, respectively. **(C)** Disease-associated *TRIP11* mutations have no effect on cellular proliferation of human fibroblast-derived chondrocytes (FDCs) from days 1 to 7 (d1-d7) of the trans-differentiation protocol. No significant differences are found between ACG1A (case A2 and A1, n=2), ODCD (cases 6 and 3, n=2) patient-derived primary cells and matched controls (CTL_1 to 4, n=4). Data points represent the mean quadruplicate cell countings, error bars represent standard deviation. **(D)** As determined by active caspase-3 enzyme-linked immunosorbent assay (ELISA), apoptosis remains constantly low in human FDC cells undergoing trans-differentiation. Data in (C) and (D) represent mean ± SD of quadruplicates (n=4, two-way ANOVA with Bonferroni's post-test); ns., not statistically significant. **(E)** Real-time quantitative reverse transcription PCR (RT-qPCR) analysis of *COL10A1* and **(F)** *MMP13* using cDNA derived from control (CTL_1 and 2) and patients' FDC cells (ACG1A cases A1, A2 and ODCD cases 6, 3). *TBP* and *HPRT* were used for normalization. The average value of the controls (n=12) was set to 1. For *TRIP11*-mutant cells, data points represent the mean of quadruplicates (n=4), error bars indicate SD. Statistical differences were assessed by two-way ANOVA with Bonferroni's post-test; **** $p<0.0001$, ns., not significant.

Supplemental Table 2: *TRIP11* sequencing primers.

Forward-Primer	Sequence 5'→3'	Reverse-Primer	Sequence 5'→3'
TRIP11-Ex1.1F	CGATCCCAGCCTCCCTTG	TRIP11-Ex1.1R	CATCGCGGCGAGTTTAGAGAAC
TRIP11-Ex1.2F	CTGCGTTTCTAGGCAGAACCTG	TRIP11-Ex1.2R	TCGACTTAACGTAGGAGCAGTTTCAC
TRIP11-Ex2F	TCTTCTGAATGCTTTCATATCAGCCTAC	TRIP11-Ex2R	AGTGCTAAGCAGTACATTTAAAAAGAAAGG
TRIP11-Ex3F	GGCCTTTTCTTAGCAGAAAATCAATC	TRIP11-Ex3R	TCAGAAATTATAAGCAACAGATCCCTAAAG
TRIP11-Ex3FS	CTGACCTCATGATCTGCCTTGG		
TRIP11-Ex4F	GTAACATTATTTATGCTTTAATTGTGAGTGTG	TRIP11-Ex4R	TTCCTAGTCCAGGGTTCTTTCTG
TRIP11-Ex5F	TGGCATTCTTTAGTCGGAATCTG	TRIP11-Ex5R	AACTGAGACTTTTACTAATTCAGGCCTATG
TRIP11-Ex6F	AGAATTCTTTTTAATAATGCACACTTTTGG	TRIP11-Ex6R	GGGCAAGTTTACAGCACTGTCC
		TRIP11-Ex6RS	CCAGCTACTTGGGAGGCTGATG
TRIP11-Ex7F	TTTAAGCTTTCCAAATTCTGAACTGTC	TRIP11-Ex7R	CACAATTCTACCCAAATGACACACAG
TRIP11-Ex8F	CTGTTACTGCTTGCCAGTGGTG	TRIP11-Ex8R	TGAAACCAGTCATGCATTTTCG
TRIP11-Ex9F	GAGTGTCGAAAATGCATGACTGG	TRIP11-Ex9R	TTCCACCTAGCCTGAAAAGCATC
TRIP11-Ex9FS	CCGTGTCTGGCCAAGTATTGC		
TRIP11-Ex10F	AGCACTTAAATAGAATCCCAAGTTTTGC	TRIP11-Ex10R	AACATGGTCACACTCATTTGTTGG
TRIP11-Ex11.1F	TGTTTTTAAGAAGGATTAGAAATATTTGAGG	TRIP11-Ex11.1R	TTTCATTGAGCTGTTCTAATTCTGAAA
TRIP11-Ex11.2F	AAGGAGCATATTAGACAAAATGAGGAG	TRIP11-Ex11.2R	TTGTTCAATATTCTTTTTGAGTTCTGC
TRIP11-Ex11.3F	ACCATTGAAGAACTGTCAAATGCAC	TRIP11-Ex11.3R	TTCATTTGCTCATGCTGGTATC
TRIP11-Ex11.4F	AGAGTCGAACCGCACCTGT	TRIP11-Ex11.4R	CATTTTGTGATATTCTGTTTTTAGATGG
TRIP11-Ex11.5F	CARCAGCAACTGCAGGCTTATG	TRIP11-Ex11.5R	GACTGGGGAGTAAGCAATGATGC
TRIP11-Ex11.6F	CCTGATSCAAAGTTATGAGCAGAATG	TRIP11-Ex11.6R	TTSTTACTGCCTGCCTCAAAAG
TRIP11-Ex11.7F	TGCCAYCTCAGAGCTRGAAAG	TRIP11-Ex11.7R	AACAATTTGAGGGAGAAYTTCCAGATG
TRIP11-Ex12F	CTTTTTGTTGATTTGGGGGTTC	TRIP11-Ex12R	CTTTATGGGTGGGCAATTTTTC
TRIP11-Ex12FS	TTTTTAAATTAACTAGGAGTCTCTCTGAGC		
TRIP11-Ex13F	TGGGAAAAAGATTTGATAACAAAATGG	TRIP11-Ex13R	TGTGAAACCCCCACACACACTC
TRIP11-Ex14F	GGATGGTAGTTTTCAAGGGAAAGG	TRIP11-Ex14R	TGATCATCTTTCCTTGTATTTCCTCAAC
TRIP11-Ex14FS	ATAAAATAAAGTAATAAGATGTAGGGAAGGTG		
TRIP11-Ex15F	GGAARGTGATCTCATTAGTTTAGWACC	TRIP11-Ex15R	GCTACAGCAGAGGGCTGGATTC
TRIP11-Ex16F	AAAGAATGTGGGTTCTGCCTTTG	TRIP11-Ex16R	GGTCTCACTCAACTATGCCAATTTATG
TRIP11-Ex17F	TGCACTCTGGCTTTTAGAGGATTTC	TRIP11-Ex17R	CCTCATTAAAGCTGCATCCTTCC
TRIP11-Ex18F	AAAGATACACATTTTGGGATGTTTG	TRIP11-Ex18R	TGGGGATATTTCAACATTTCAGC
TRIP11-Ex19F	CATTTACTAGGACTTGAACTTGAAAAGCA	TRIP11-Ex19R	AAGACTGCCTATGGCAGCATGA
TRIP11-Ex19FS	TTTKTCTGTTTTRAAATAATCTTTCAGTTG	TRIP11-Ex19RS	TTCTTACACTRTYAACCACAGATTGC
TRIP11-Ex20F	AAAAGTGCCAGTATTTTCAATTGTTC	TRIP11-Ex20R	GCCTGATGCCTTATTGGAATTCTG
TRIP11-Ex21F	TCAAGGGAGAATTTTTAAGTTGATAAGTG	TRIP11-Ex21R	TTCAGAGAAAGCATAATTGCGAAC

Supplemental Table 3: Primer used for cloning of the *TRIP11* constructs.

**Primer**	**Sequence 5'-3'**	
TRIP11DeltaExon9short_F	Forward	ggggtaccAGAATTAAGTGACTATGA
TRIP11DeltaExon9short_F2	Forward	AGAATTAAGTGACTATGAAGAACGAATTG
TRIP11DeltaExon4and9_R	Reverse	GGTTCGACTCTGCTCTTCCC
TRIP11DeltaExon4_F	Forward	ttggtaccATGTCGTCCTGGCT

Supplemental Table 4: Primer used for sequencing of the *TRIP11* constructs.

Primer	Sequence 5'-3'	
CMVPrimingsite	Forward	CGCAAATGGGCGGTAGGCGTG
TRIP11_seq_F1	Forward	AGTCTCTGGGTCAAGTCGGG
TRIP11_seq_F5	Forward	GAAGAGCAGAGTCGAACCGC
TRIP11_seq_F9	Forward	CATCAAGCCAGTGTGCAGGT
TRIP11_seq_F11	Forward	CACCTTTGCCAGCGTTACCT
TRIP11_seq_F13	Forward	TGAACATGCCACTTCCTGCC
TRIP11_seq_F15	Forward	GGAGAATTGCTTGACCCCGG
TRIP11_seq_F16	Forward	TGAATTCCACCTTCGCTGCC
TRIP11_seq_F2	Forward	TGTACCAGCAACCACTGCAT
TRIP11_seq_R3	Reverse	TCACCTGCTGCTTCCACTCT
TRIP11_seq_F3	Forward	TTGCCCAGAGTGCATCAGTG
TRIP11_seq_F4	Forward	AGCATGTGAAGATGTGAGGCA
TRIP11_seq_F7	Forward	CCCAGCTGTCTTCTGCATCA
TRIP11_seq_F8	Forward	CCAAGTCATGTTGGCCCTGA
TRIP11_seq_R4	Reverse	CAACTGCTCCATCTCCTCCC
TRIP11_seq_F10	Forward	AGGGAGGAGATGGAGCAGTT
TRIP11_seq_F12	Forward	GACTTTGTGGAGACAGGCCA
TRIP11_seq_F14	Forward	AACTTGTCCGCCTCTCATCC
TRIP11_seq_R5	Reverse	AAACCATGGGATGAGAGGCG
TRIP11_seq_F6	Forward	TCCAGCACTGGTAATGAGGC
TRIP11seq-R1	Reverse	AGCCCAACACAATTCTGCCT
TRIP11seq-R2	Reverse	TCCTCCTGCAGCTTTGAACT

Supplemental Table 5: Primer used for RT- and RT-qPCR.

Primer	Sequence 5'-3'	
TRIP11_F3/R2_qPCR<br>(Thyroid Hormone Receptor Interactor 11)	Forward<br>Reverse	GCGTCATGAAGTGTTACGGT<br>AGTCATCCACCTGGTAACAC
TRIP11_F3/R3<br>(Thyroid Hormone Receptor Interactor 11)	Forward<br>Reverse	GCGTCATGAAGTGTTACGGT<br>GGTGGAATGGATGGATGAGA
HPRT1<br>(Hypoxanthine Phosphoribosyl-transferase1)	Forward<br>Reverse	GCAGACTTTGCTTTCCTTGGTCAG<br>CAAGCTTGCGACCTTGACCATC
TBP<br>(TATA Box Binding Protein)	Forward<br>Reverse	TGTGCTCACCCACCAACAATTTAG<br>TTTCTGCTCTGACTTTAGCACCTG
TRIP11_E7E11<br>(Thyroid Hormone Receptor Interactor 11)	Forward<br>Reverse	GAGTGCATCAGTGGAAGAAGTG<br>CCAATTCTTCTATCTCAGCTATCA
TRIP11_E1E5<br>(Thyroid Hormone Receptor Interactor 11)	Forward<br>Reverse	ATATGCTGATGGAGGGCACG<br>TCTGTTCCTTGTGCTTTGGAAG
TRIP11 _E14E19<br>(Thyroid Hormone Receptor Interactor 11)	Forward<br>Reverse	CCTGCAGATGGTACTAGAGC<br>AGTCATCCACCTGGTAACAC
TRIP11_E1E11<br>(Thyroid Hormone Receptor Interactor 11)	Forward<br>Reverse	ttggtaccATGTCGTCCTGGCT<br>GGTTCGACTCTGCTCTTCCC
COLX	Forward<br>Reverse	ATGATGAATACACCAAAGGCTACCT<br>AGCCACACCTGGTCATTTTCT
mIFT20TOPO	Forward<br>Reverse	ACCTCCTGATTGCCACTGTC<br>TAACACTGCATGCGCACATC
M13	Forward<br>Reverse	GTAAAACGACGGCCAG<br>CAGGAAACAGCTATGAC
